# Repurposing anti-viral subunit and mRNA vaccines T cell immunity for intratumoral immunotherapy against solid tumors

**DOI:** 10.1038/s41541-025-01131-y

**Published:** 2025-04-25

**Authors:** Shiv K. Sethi, Claire E. Bradley, Lukas Bialkowski, Yuk Ying Pang, Cynthia D. Thompson, John T. Schiller, Nicolas Çuburu

**Affiliations:** 1 National Cancer Institute, NIH, Bethesda, MD USA; 2 Present Address: Beckman Coulter, Bethesda, USA

**Keywords:** Tumour immunology, Adjuvants, Peptide vaccines, Protein vaccines, RNA vaccines, Cancer microenvironment, Cancer immunotherapy

## Abstract

Intratumoral (IT) immunotherapy can stimulate the tumor microenvironment and enhance anti-tumor immunity. We investigated IT delivery of three licensed viral vaccines—Shingrix (VZV shingles), Gardasil-9 (HPV), and Spikevax (SARS-CoV-2)—in prevaccinated mice using the murine tumor model TC-1, which expresses HPV16 oncogenes E6 and E7. Shingrix IT injection often induced tumor regression and resistance to secondary challenge. Injecting a VZV glycoprotein E (gE)-derived MHC-II-restricted peptide with polyI:C also led to durable remission, highlighting the role of gE-specific CD4^+^ T cells. While Gardasil-9 IT injection alone was ineffective, combining a HPV L1-derived MHC-I-restricted peptide with polyI:C or Shingrix enhanced tumor regression. Both approaches elicited CD8^+^ T cells against the E7 tumor viral oncoprotein. Tumor microenvironment analysis revealed remodeling of the myeloid compartment, significant induction of IFN-γ, TNF-α, and CXCL9 and broad gene expression reprograming. In a dual-flank model, IT injection of Shingrix with an MHC-I-restricted E7 tumor-specific peptide eliminated primary and non-injected tumors. Finally, Spikevax IT injection showed modest tumor growth delay, while improved control was observed with a SARS-CoV-2 spike-derived MHC-I-restricted peptide and polyI:C. These results demonstrate the potential of licensed vaccines as promising platforms for IT immunotherapy, either alone or combined with vaccine- or tumor-derived MHC-I-restricted peptide epitopes.

## Introduction

Cancer immunotherapy has become a standard of care and first-line treatment for several types of solid tumors including melanoma, lung, and colon cancer^1^. While systemic immunotherapy has led to remarkable cancer remission, it is often associated with high rates of immune-related systemic toxicity or resistance in the tumor microenvironment (TME). Consequently, local cancer immunotherapy against solid tumors has emerged as a potentially safer and more effective approach to stimulate the tumor immune microenvironment and promote disseminated anti-tumor immune responses^2^.

Intratumoral (IT) drug delivery has been extensively investigated in preclinical and clinical settings^3^. This approach aims to activate cytotoxic T cells, improve antigen presentation, and reshape the tumor immune microenvironment through inhibition of immunosuppressive cells and/or activation of immune-enhancing cells. Intratumoral agents used for local immunotherapy include cytokines, chemokines, pattern recognition receptor agonists, oncolytic viruses, and bacteria^4^.

Live-attenuated vaccines have been assessed for their oncolytic potential and intrinsic ability to stimulate an innate immune response^5^. Licensed vaccines present the advantage of being off-the-shelf reagents with an established safety profile that could be deployed rapidly in low-resource settings, as well as used in combination with other therapeutic modalities. However, whether widespread immunity against live-attenuated vaccines could diminish anti-tumor responses or whether induction of neutralizing antibodies prevents effective boosting of the initial responses has not been fully addressed^5–7^.

Subunit vaccines have emerged as a safer and efficacious alternative to live-attenuated or inactivated pathogen vaccines. They have been successfully implemented against viruses such as hepatitis B virus, human papillomavirus (HPV) and shingles caused by varicella zoster virus (VZV) reactivation^8^. Subunit vaccines are generally composed of virion-derived protein antigens and molecular adjuvants to potentiate immunogenicity. Indeed, the selection of appropriate adjuvants is crucial for developing effective vaccine immunity^8^. While aluminum salts (alum) have been widely used to improve bioavailability of vaccine antigens and activate the NOD-like receptor protein 3 (NLRP3) inflammasome, they are associated with dominant T helper 2 (Th2)-type responses^9^. New classes of adjuvants targeting the innate immune system have been incorporated in subunit vaccines to elicit T helper 1 (Th1)-type responses and CD8^+^ T cell responses. For instance, HPV L1 capsid protein-based virus-like particle (VLP) vaccines intrinsically induce CD4^+^ Th1 and CD8^+^ cytotoxic T cell responses which are improved with the addition of the Toll-like receptor 4 (TLR4) agonist monophosphoryl lipid A (MPLA)^10,11^. Similarly, the shingles vaccine, based on the VZV glycoprotein envelope E (gE) and AS01B, a liposome-based adjuvant incorporating MPLA and saponin QS-21, has demonstrated potent immunogenicity and a strong bias toward CD4^+^ Th1-type responses, even in older individuals^12,13^.

The recent worldwide implementation of mRNA vaccines against severe acute respiratory syndrome coronavirus 2 (SARS-CoV-2) during the coronavirus disease 2019 (COVID-19) pandemic has provided the impetus for developing mRNA vaccines against other infectious diseases and cancer^14^. Immunogenicity studies of mRNA vaccines indicate a bias toward Th1-type responses and the potent induction of CD4^+^ and CD8^+^ T cell responses^15^. mRNA vaccines formulated with lipid nanoparticles are inherently immunogenic, causing lipid-mediated inflammasome activation and the recognition of mRNA molecules by melanoma differentiation-associated protein 5 (MDA-5)^16,17^. The mRNA SARS-CoV-2 vaccines have shown a bias toward Th1-type responses supported by a transient increase in chemokines CXCL9/10, interleukin (IL)-15 and interferon gamma (IFN-γ) in the sera of vaccinated individuals^18^.

Our previous study in murine syngeneic tumor models demonstrated that preexisting CD4^+^ and CD8^+^ T cells against a chronic murine cytomegalovirus (MCMV) infection could be activated in a suppressive tumor immune microenvironment by intratumoral injection of cognate CD4^+^ and CD8^+^ T cell peptide epitopes^19^. This approach led to local activation, recruitment and expansion of MCMV-specific CD8^+^ T cells, immediate debulking of the tumor mass and broad TME activation in immunologically cold tumors. Notably, local activation of MCMV-specific CD4^+^ T cells led to immune activation in the TME and promoted epitope spreading against tumor-specific antigens.

Building on these findings, we sought to investigate whether anti-viral vaccine T cell immunity could be harnessed by intratumoral delivery of subunit vaccines and exert tumor suppressive effects in the syngeneic tumor model TC-1, driven by the viral oncogenes E6 and E7, and H-ras^20^. We assessed the modulation of the TME, tumor control, and induction of antitumor immunity in primary subcutaneous tumors, and abscopal responses in a dual flank model. Specifically, we assessed two subunit vaccines against HPV and VZV, chosen for their propensity to preferentially elicit CD8^+^ and CD4^+^ T cell responses, respectively. In prevaccinated animals, we assessed intratumoral injection of the licensed vaccines alone or combinations of selected MHC-I- and MHC-II-restricted epitopes derived from HPV16 L1 or VZV gE, respectively. Finally, we assessed the tumor suppressive effect of a licensed SARS-CoV-2 mRNA vaccine and an MHC-I-restricted spike immunodominant epitope after intratumoral delivery and the recall of anti-SARS-CoV-2-specific CD8^+^ T cells.

## Results

### Differential propensities of VZV and HPV subunit vaccines to elicit CD4^+^ and CD8^+^ T cell responses and broad cytokine and chemokine production

We have previously demonstrated that intratumoral injection of viral epitopes could redirect preexisting CD4^+^ and CD8^+^ T cell responses against a latent virus infection to control tumor progression and induce epitope spreading against a tumor-specific antigen^19^. In this study, we sought to determine whether CD4^+^ and CD8^+^ T cell responses elicited by subunit vaccines could be harnessed in the immune-suppressed TME for anti-tumor therapy. Subunit vaccines have a narrow range of epitopes and induce skewed immune responses; therefore, we selected two licensed subunit vaccines against VZV reactivation (Shingrix, hereafter VZV-vax) and HPV (Gardasil-9, hereafter HPV-vax) which have demonstrated remarkable protection in humans. While VZV-vax is skewed toward CD4^+^ Th1 responses, HPV-vax elicits CD4^+^ Th1 and CD8^+^ T cell response in humans^11,21,22^.

**Fig. 1:**
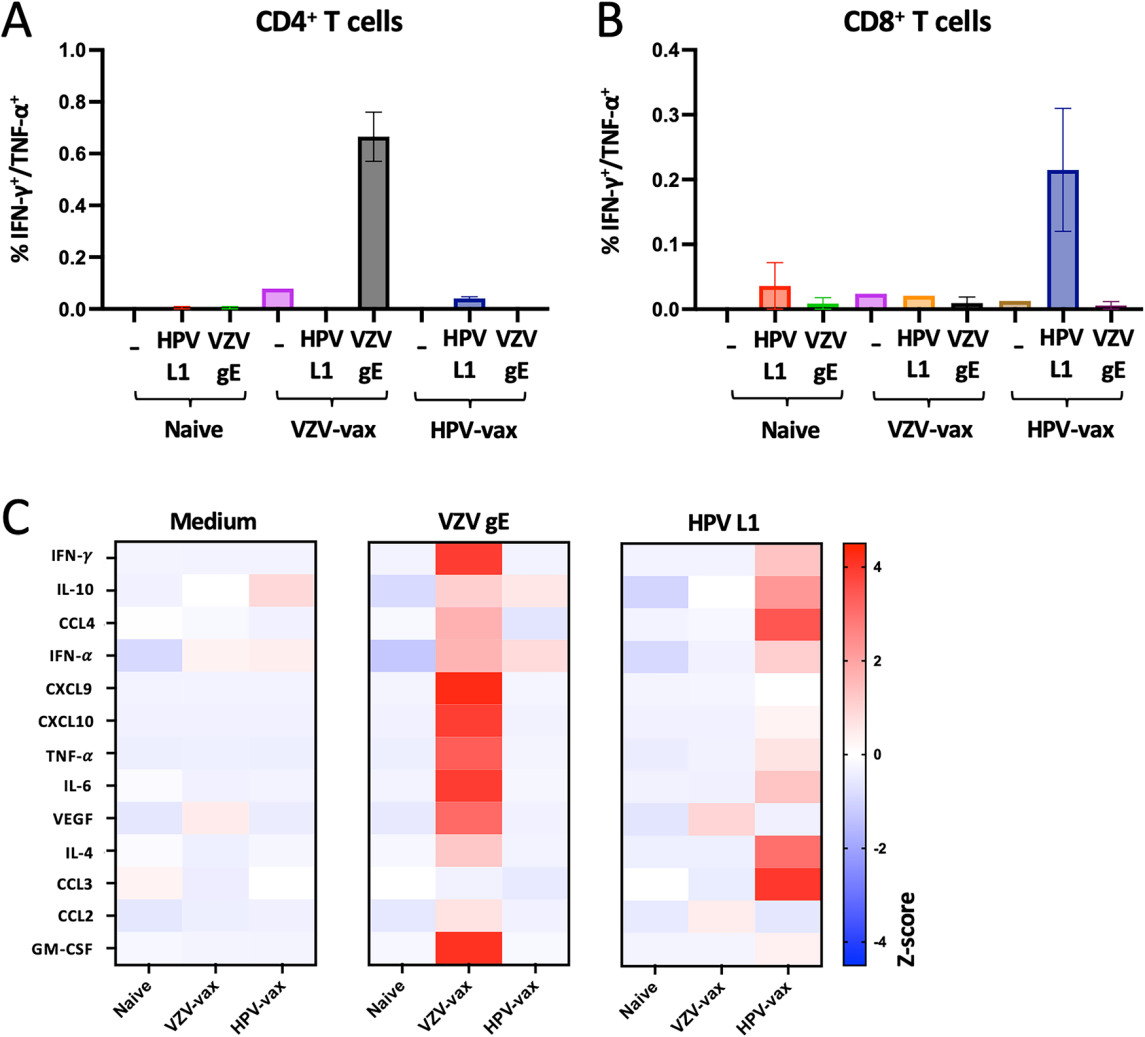
Differential propensities of VZV and HPV subunit vaccines to elicit CD4^+^ and CD8^+^ T cell responses in C57BL/6 mice. Splenocytes from naïve, VZV-vax and HPV-vax immunized mice following an intramuscular prime-boost regimen were analyzed after in vitro stimulation with overlapping peptide libraries derived from VZV gE and HPV L1 antigens or medium only. **A**, **B** Production of IFN-γ and TNF-α by (**A**) CD4^+^ and (**B**) CD8^+^ T cells was analyzed by flow cytometry and intracellular cytokine staining. Data are shown as mean percentage of positive CD4^+^ or CD8^+^ T cells ± standard error of the mean (SEM). **C** IFN-γ, IL-10, CCL4, IFN-α, CXCL9, CXCL10, TNF-α, IL-6, VEGF, IL-4, CCL3, CCL2, and GM-CSF production was measured using a bead-based multiplex immunoassay in supernatants of splenocyte cultures stimulated for 48 h with overlapping peptide libraries derived from the VZV gE and HPV L1 antigens. Heatmap represents the mean Z-score of the amount of each analyte in pg/ml in supernatants (*n* = 3 per group).

We immunized C57BL/6 mice following a prime-boost regimen two weeks apart and analyzed the CD4^+^ and CD8^+^ T cell responses two weeks after the last immunization. Splenocytes from immunized and naïve control mice were stimulated with medium, VZV gE or HPV16 L1 overlapping peptide libraries (OPL), and cytokine production by CD4^+^ and CD8^+^ T cells was analyzed by flow cytometry and intracellular cytokine staining (Fig. 1A, B, and Supplementary Fig. 1A and B). IFN-γ and tumor necrosis factor (TNF)-α production was pronounced in CD4^+^ T cells (Fig. 1A) but not in CD8^+^ T cells (Fig. 1B) after restimulation with VZV gE OPL in VZV-vax immunized mice. In contrast, HPV-vax immunization induced specifically the production of IFN-γ and TNF-α by CD8^+^ T cells in response to HPV16 L1 OPL stimulation (Fig. 1B) but not by CD4^+^ T cells (Fig. 1A).

Next, we analyzed the production of major cytokines and chemokines in spleen culture supernatants after a 48-hour restimulation with gE and L1 OPL using a multiplex immunoassay. Both VZV-vax and HPV-vax induced a broad cytokine and chemokine response after in vitro restimulation with gE and L1 OPL, respectively (Fig. 1C). The production of IFN-γ and TNF-α in culture supernatants of VZV gE and HPV L1 stimulated cultures, but not in medium-only conditions, confirmed the cytokine production observed at the cellular level by intracellular cytokine staining (ICCS). Interestingly, the production of the IFN-γ-induced chemokines CXCL9 and CXCL10 was pronounced in cultures from VZV-vax immunized mice restimulated with VZV gE OPL but more modest in cultures from HPV-vax immunized mice restimulated with HPV L1 OPL (Fig. 1C). Granulocyte-monocyte colony stimulating factor (GM-CSF) production was notable in VZV-restimulated cultures but not in HPV L1-restimulated cultures (Fig. 1C), suggesting either direct production by CD4^+^ T cells or by accessory cells activated in the culture. In contrast, in HPV L1 OPL restimulated cultures, but not in VZV gE, we observed a robust production of CCL3 which is typically produced by CD8^+^ T cells and is involved in the recruitment of both lymphoid and myeloid cells in solid tumors.

### Intratumoral injection of an HPV16 L1-derived MHC-I-restricted minimal peptide epitope with poly I:C, but not HPV-vax, confers tumor control, induces epitope spreading and reprograms the tumor microenvironment in pre-immunized mice

**Fig. 2:**
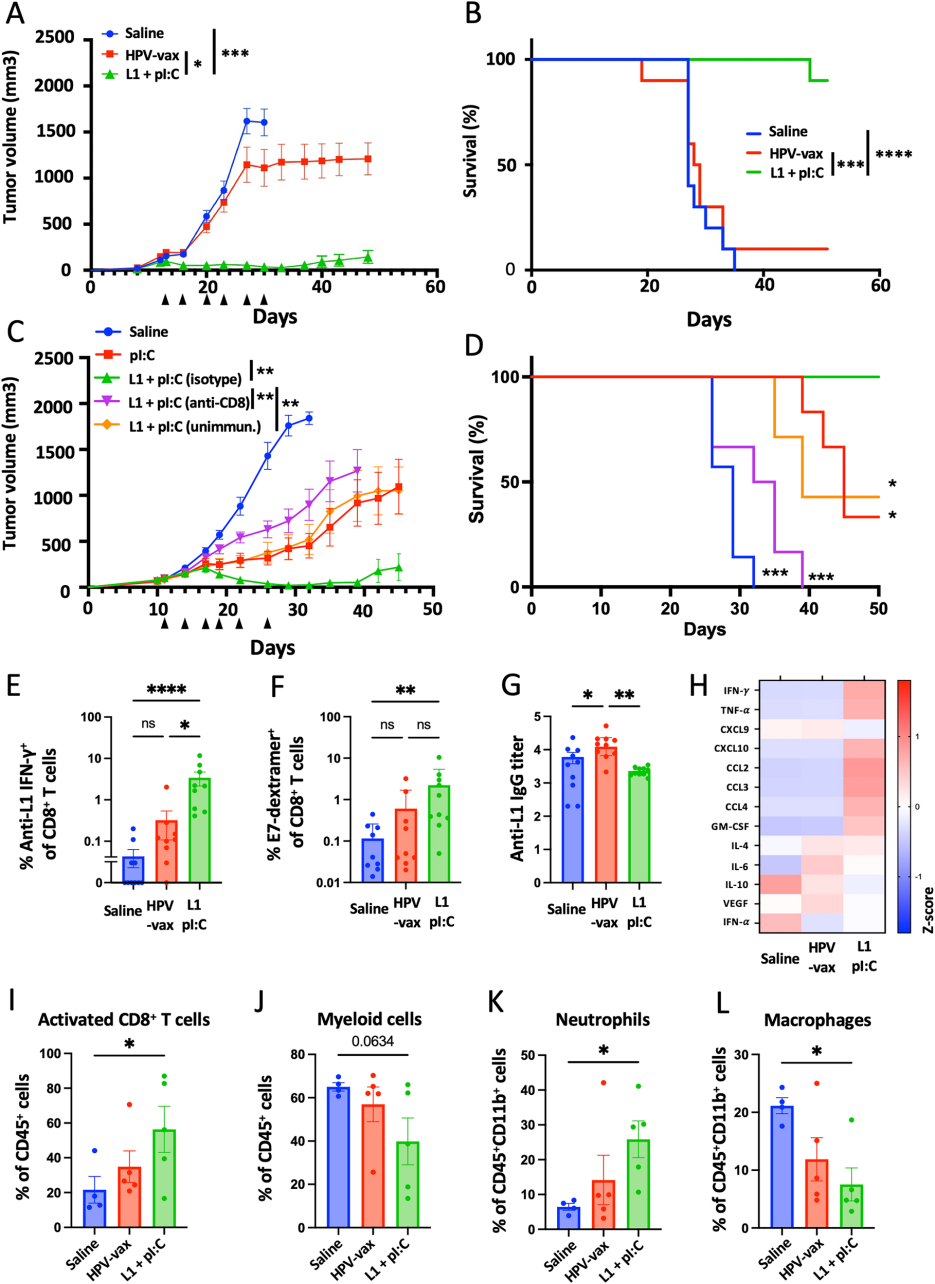
IT injection of an L1-derived MHC-I-restricted peptide with poly I:C but not HPV-vax confers tumor control, induces epitope spreading, and reprograms the tumor microenvironment. Experimental design: C57BL/6 mice were prime-boost immunized intramuscularly with the HPV vaccine or unimmunized prior to TC-1 tumor subcutaneous challenge one week after boost. When tumor reached a volume between 50 and 100 m^3^, mice were treated six times with saline, HPV-vax alone (1/25^th^ of the human dose) or L1_165-173_ peptide (0.5 μg) admixed with polyI:C LMW (50 μg). Mice were monitored twice a week for (**A**, **C**) tumor growth and (B, D) survival. **A**, **C** Tumor growth is shown as the mean of tumor volume for each group and SE (*n* = 6–9 per group). Statistical analysis with Dunn’s test was performed for multiple comparison of tumor growth. **B**, **D** Survival comparisons were assessed by Mantel–Cox test (****P < 0.0001, ***P < 0.001, **P < 0.01 *P < 0.05 n.s.: not significant). **E** IFN-γ production was assessed after IT treatment by intracellular staining of circulating CD8^+^ T cells after in vitro restimulation with L1_165-173_ peptide. Data are shown as individual values and mean of cytokine production of CD44^+^CD8^+^T cells with SE. **F** Circulating anti-tumor E7-specific CD8^+^ T cell responses were measured by dextramer staining after IT treatment. Data are shown as individual percentage and mean of H2-D^b^/E7_49-57_^+^CD44^+^CD8^+^ T cells. **G** anti-HPV16 L1 VLP antibody responses were measured by ELISA in plasma samples. Data are shown as individual and geometric mean of IgG EC50 titer and 95% confidence interval. **H** Cytokine and chemokine production was measured using a bead-based multiplex immunoassay in tumor lysates. Heatmap shows the average Z-score of the amount of each analyte per 50 μg of protein. **I**–**L** The cellular infiltrate was assessed by flow cytometry. Data are shown as individual values and mean percentage within CD45^+^ cells of (**I**) activated CD8^+^ T cells defined as CD8^+^CD44^+^CD62L^-^PD1^+^, (**J**) myeloid cells defined as CD11b^+^, and percent within CD11b^+^ cells of (**K**) neutrophils defined as CD11b^+^Ly6G^+^Ly6C^int^ and (**L**) macrophages defined as CD11b^+^F480^+^Ly6G^-^Ly6C^int^. Data are shown as individual values and mean percentage of cells within indicated gated population and SE. Statistical analysis (*P < 0.05 or numeric values) was performed using Dunn’s test for multiple comparison between groups (*n* = 4–5 per group).

We sought to determine whether intratumoral injection of HPV L1 vaccine antigens could confer tumor protection in mice pre-vaccinated with the HPV-vax (equivalent to 1/10^th^ of the human dose). C57BL/6 mice were prime-boost immunized, and one week after the last immunization, mice were implanted subcutaneously with TC-1 tumor cells which express the HPV16 viral oncogenes E6 and E7, as well as H-ras^20^. Once the tumors reached a volume between 50 and 100 mm^3^, we proceeded to the IT injection of HPV-vax (1/25^th^ of the human dose) or an immunodominant MHC-I-restricted minimal peptide epitope derived from HPV16 L1 (L1_165-173_, 0.5 μg) admixed with low molecular weight (LMW) polyinosinic-polycytidylic acid (polyI:C, 50 μg), twice a week for 6 consecutive intratumoral injections following a schedule established previously^19^. The IT injection of the HPV-vax alone did not confer tumor control as measured by tumor growth and mean survival endpoint, defined as a tumor volume of 1500 mm^3^, (MS = 28.5 days) compared to the saline-treated control group (MS = 28.0 days) (Fig. 2A, B). In contrast, IT injection of the MHC-I-restricted L1_165-173_ minimal peptide epitope together with polyI:C led to pronounced tumor control and significantly improved survival compared to saline-treated and HPV-vax treated mice (Fig. 2A, B).

Next, we assessed the role of pre-existing anti-vaccine CD8^+^ T cells to the antitumor response observed after intratumoral injection of polyI:C and the L1_165-173_ minimal peptide epitope (Fig. 2C, D). While polyI:C alone delayed tumor growth as previously described in other models^23–26^, only the combination with the L1_165-173_ minimal peptide epitope led to complete tumor clearance with 4 out of 6 mice tumor-free (Fig. 2C). Antibody-mediated depletion of CD8^+^ T cells during treatment with polyI:C combined with the L1_165-173_ minimal peptide epitope abrogated protection (Fig. 2D), indicating a critical contribution of CD8^+^ T cells to the protective anti-tumor response (Fig. 2C, D). Finally, mice that were not immunized with the HPV-vax prior to treatment showed similar protection compared to polyI:C alone, suggesting that initial recall of preexisting CD8^+^ T cells induced by vaccination substantially contributed to the protective anti-tumor response.

Then we assessed the anamnestic response of L1-specific CD8^+^ T cells after repeated IT injection of either the HPV-vax or the L1_165-173_ peptide epitope with polyI:C (Fig. 2E). Blood leukocytes collected after the last IT treatment were restimulated in vitro with the immunodominant L1_165-173_ peptide, and L1-specific IFN-γ-producing CD8^+^ T cells were analyzed by flow cytometry and intracellular staining (Supplementary Fig. 2A and B). Intratumoral injection of HPV-vax led to a modest increase in the percentage of IFN-γ^+^ CD8^+^ T cells; in contrast, we observed a significant increase of an order of magnitude in the percentage of L1-specific IFN-γ^+^ CD8^+^ T cells after IT injection of the L1 immunodominant peptide with polyI:C (Fig. 2E). This suggests that the L1_165-173_ immunodominant minimal peptide epitope is efficiently presented upon IT injection but not when derived from the HPV-vax VLP. Notably, IT treatment with L1_165-173_ peptide and polyI:C expanded circulating CD8^+^ T cells directed against the tumor-specific viral oncoprotein E7 as measured by dextramer staining, but not in saline- or HPV-vax-treated groups (Fig. 2F, Supplementary Fig. 2C and D). High IgG anti-L1 titers were measured after prime-boost immunization of the saline-treated group, which were increased in the group treated IT with the HPV-vax, but not in the group treated with L1_165-173_ peptide and polyI:C (Fig. 2G), suggesting that this peptide is not recognized by L1 VLP-specific B cells and that preexisting anti-HPV16 VLP antibodies may not interfere with its MHC-I binding and presentation to CD8^+^ T cells.

Next, we sought to characterize whether IT injection of the HPV16 L1-derived peptide epitope with polyI:C could trigger immune activation in the TME and induce Th1 cytokines and chemokines. We used a multiplex immune panel designed to measure response to cancer immunotherapy treatments, which includes CCL2, IL-10, CCL4, IFN-α, CXCL9, CXCL10, TNF-α, IL-6, vascular endothelial growth factor (VEGF), IL-4, CCL3, IFN-γ, and GM-CSF, to analyze lysates from tumors collected 36 h after the last IT treatment (Fig. 2H). Intratumoral injection of HPV-vax did not induce changes in cytokine and chemokine production in tumor lysates compared to the saline-treated group (Fig. 2H). In contrast, IT injection of the immunodominant HPV16 L1 minimal peptide epitope with polyI:C was associated with an increase in IFN-γ and TNF-α compared to the saline- and HPV-vax-treated groups, suggesting local activation of CD8^+^ T cells (Fig. 2H). In addition, the IT L1 peptide/polyI:C-treated mice displayed an increase in CXCL10, CCL2, CCL3 and CCL4 and GM-CSF in the tumor lysate which could be involved in the recruitment, retention and maturation of myeloid and lymphoid cells in the tumor (Fig. 2H). In fact, flow cytometry analysis of the cellular lymphoid and myeloid tumor infiltrate showed that IT injection of the L1 minimal peptide epitope with polyI:C led to an increase in activated CD8^+^ T cells (CD45^+^CD4^-^CD8^+^CD44^+^CD62L^-^PD1^+^) (Fig. 2I, Supplementary Fig. 3A), a non-significant decrease in myeloid cells (CD45^+^CD11b^+^)(Fig. 2J, Supplementary Fig. 3B), an increase in neutrophils (CD45^+^CD11b^+^Ly6C^mid^Ly6G^+^)(Fig. 2K) and a decrease in macrophages (CD45^+^CD11b^+^Ly6C^-^Ly6G^-^ F4/80^+^) (Fig. 2L) compared to saline- and HPV-vax-treated groups.

### IT injection of VZV-vax or a VZV gE-derived MHC-II-restricted minimal peptide epitope with polyI:C confers tumor control and long-term protection

Given the potent CD4^+^ T cell response elicited by VZV-vax, we sought to determine whether IT administration of the licensed vaccine could confer tumor control. Additionally, we assessed the anti-tumor properties of a gE-derived MHC-II-restricted peptide epitope (gE_71-90_), previously identified in the C57BL/6 genetic H2^b^ haplotype^27^, in combination with polyI:C. C57BL/6 mice were immunized in a prime-boost regimen by intramuscular injection of the licensed VZV-vax (equivalent to 1/10^th^ of the human dose). One week after the final immunization, mice were challenged subcutaneously with the TC-1 tumor cells. When tumor reached a volume between 50 and 100 mm^3^, we proceeded with repeated injection of the tumor nodules with either saline, VZV-vax (equivalent to 1/40^th^ of the human dose) or a mixture of gE_71-90_-peptide (2 μg) with LMW polyI:C (50 μg). Tumors were injected twice a week for a total of six injections following a previously established schedule^19^.

**Fig. 3:**
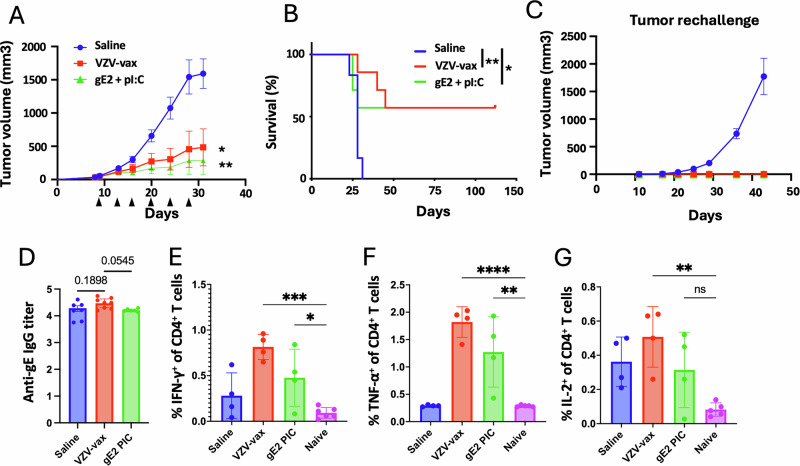
Intratumoral injection of VZV-vax or a gE-derived MHC-II-restricted peptide with polyI:C provides tumor control and long-term protection. Experimental design: C57BL/6 mice were prime-boost immunized intramuscularly with the VZV vaccine prior to TC-1 tumor subcutaneous challenge one week after boost. When tumor reached a volume between 50 and 100 m^3^, mice were treated six times with saline, VZV-vax alone (1/40^th^ of the human dose) or the MHC-II-restricted gE_71-90_ peptide (2 μg) admixed with polyI:C LMW (50 μg). Mice were monitored twice a week for (**A**) tumor growth and (**B**) survival. **A** Tumor growth is shown as the mean of tumor volume for each group and SE (*n* = 7 per group). Statistical analysis with Dunn’s test was performed for multiple comparison of tumor growth. **B** Survival comparisons were assessed by Mantel–Cox test (**P < 0.01, *P < 0.05). **C** Tumor-free mice from the VZV-vax and gE_71-90_ + polyI:C-treated groups, and naïve mice were rechallenged with TC-1 cells, 60 days after the primary challenge and tumor growth was monitored twice a week until endpoint. **D** anti-VZV gE protein responses were measured by ELISA in plasma samples. Data are shown as individual values and geometric mean of the EC50 of IgG titer and 95% confidence interval. In a separate experiment, (**E**) IFN-γ, (**F**) TNF-α and (**G**) IL-2 production was assessed 36 h after the last IT treatment by intracellular staining of circulating CD4^+^ T cells after in vitro restimulation with VZV gE overlapping peptide library. Individual cytokine production is shown as individual values and mean percentage within CD44^+^CD4^+^T cells with SE. Statistical analysis was performed using Dunn’s test for multiple comparison between groups (*P < 0.05 **P < 0.01, ***P < 0.001, ****P < 0.0001, numeric values, ns = not significant) (*n* = 4 per group).

All saline-treated tumors reached the endpoint (defined as a tumor volume of 1500 mm^3^) within 28 days. In contrast, mice treated with VZV-vax alone showed tumor growth control, with half achieving complete regression and remaining tumor-free for 112 days (Fig. 3A, B). Notably, mice treated with the gE_71-90_ MHC-II-restricted minimal peptide epitope with polyI:C also controlled growth of the primary tumor which suggests a contribution of CD4^+^ T cells to the antitumor response (Fig. 3A, B). Importantly, mice that cleared their primary tumors after IT treatment with VZV-vax or gE_71-90_ peptide with polyI:C were fully protected against a secondary tumor challenge, indicating that they had mounted protective antitumor immunity (Fig. 3C). All mice primed with VZV-vax elicited a potent anti-gE IgG response (Fig. 3D). Finally, IT injection of VZV-vax led to an increase in IFN-γ-, TNF-α- and IL-2-producing CD4^+^ T cells 36 h after the last IT injection and to a lesser extent after injection of the minimal gE_71-90_ MHC-II-restricted epitope (Fig. 3E–G).

### Combination of VZV-vax and an HPV16 L1-derived peptide induces local immune activation and immunogenic cell death of tumor cells

**Fig. 4:**
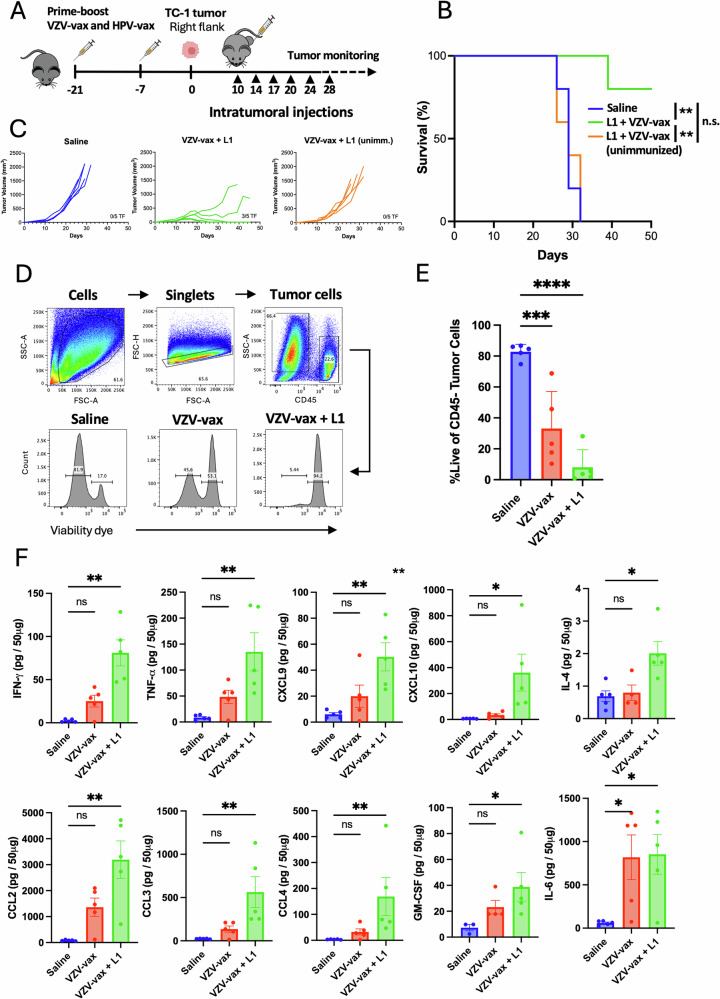
Intratumoral injection of VZV-vax and an HPV L1-derived peptide promotes tumor control and TME immune activation. Experimental design (**A**): C57BL/6 mice were prime-boost immunized intramuscularly with the VZV and HPV vaccines or left unimmunized prior to TC-1 tumor subcutaneous challenge one week after boost. When tumor reached a volume between 50 and 100 m^3^, mice were treated six times with saline or VZV-vax admixed with L1_165-173_ peptide (0.5 μg) for tumor growth (**B**) and survival (**C**), or four times with saline, VZV-vax alone (1/40^th^ of the human dose) alone or admixed with L1_165-173_ peptide (0.5 μg) for tumor cell viability (**D**, **E**) and cytokine/chemokine production in the tumor (**F**). **D** Tumor cell viability after treatment was measured 36 h after the last IT injection by flow cytometry by live/dead dye staining on CD45^-^ cells. **E** Data are shown as individual values and mean percentage of live CD45-negative cells with SE. **F** Cytokine and chemokine production was measured in tumor protein lysates using a bead-based multiplex immunoassay. Data are shown as individual values and mean amount per 50 μg of protein with SE and symbols represents individual mice. **E**, **F** Statistical analysis (*P < 0.05, **P < 0.01, ***P < 0.001, ****P < 0.0001, n.s.: not significant) was performed using Dunn’s test for multiple comparison between groups (*n* = 5 per group). **A** Tumor icon was created using Biorender.

Given the immunotherapeutic potential of VZV-vax alone and the HPV16 L1_165-173_ MHC-I-restricted peptide epitope adjuvanted with polyI:C for IT delivery, we investigated whether the VZV-vax and the L1_165-173_ peptide could be combined for IT delivery in VZV/HPV dual-vaccinated or unimmunized mice (Fig. 4A). C57BL/6 mice were immunized in a prime-boost regimen by concurrent intramuscular injection of the licensed VZV-vax and HPV-vax in opposite quadriceps or remained unimmunized. One week after the final immunization, mice were challenged subcutaneously with the TC-1 tumor cells. When tumors reached a volume between 50 and 100 mm^3^, we proceeded with repeated injection of the tumor nodules with either saline or VZV-vax (equivalent to 1/40^th^ of the human dose) admixed with the HPV16 L1_165-173_ peptide (0.5 μg). In preimmunized mice, 6 consecutive injections of TC-1 tumors with VZV-vax admixed with the L1_165-173_ minimal peptide epitope led to a pronouced delay in tumor growth and improved survival compared to saline-treated animals (Fig. 4B, C). In contrast, unimmunized mice treated with VZV-vax and the L1_165-173_ peptide were not protected compared to the saline-treated group (Fig. 4B, C). These results indicate that preexisting vaccine-induced immunity substantially contributes to the observed anti-tumor response.

Furthermore, we assessed the immune modulation of the TME in tumors treated with saline, VZV-vax, and VZV-vax admixed with the HPV16 L1_165-173_ minimal peptide epitope without polyI:C to evaluate the possibility that AS01B in VZV-vax could serve as an effective intratumoral adjuvant of MHC-I-restricted CD8^+^ T cell responses. C57BL/6 mice underwent the prime-boost vaccination regimen and TC-1 tumor challenge (Fig. 4A). When tumors reached a volume between 50 and 100 mm^3^, we proceeded with repeated injections of the tumor nodules with either saline, VZV-vax alone or VZV-vax admixed with the HPV16 L1_165-173_ peptide (0.5 μg). Tumors were injected twice a week for four consecutive injections and samples were collected 36 h after the last treatment. Tumor cell viability after treatment was assessed by flow cytometry using a fluorescent viability dye and gating on CD45^-^ cells as shown by the gating strategy and representative FACS plots (Fig. 4D). Saline-treated tumors displayed a high tumor cell viability (mean=81%, Fig. 4E). Notably, tumor cell viability was reduced after IT treatment with VZV-vax alone (mean=33%) and further reduced in the group treated with a combination of VZV-vax with the HPV16 L1_165-173_ peptide (mean=8%, Fig. 4E). Next, we assessed cytokine and chemokine production in tumor lysates using the multiplex Cytokine Release Syndrome panel (Fig. 4F). VZV-vax alone induced a modest but non-significant increase in IFN-γ, TNF-α, CXCL9, CXCL10, CCL2, CCL3, CCL4, GM-CSF and IL-6 (Fig. 4F). Cytokine and chemokine production in tumor lysates was further increased after IT treatment with VZV-vax and the HPV16 L1 peptide, suggesting that local activation of both VZV-specific CD4^+^ and HPV-specific CD8^+^ T cells could lead to broad immune activation of the local TME (Fig. 4F).

**Fig. 5:**
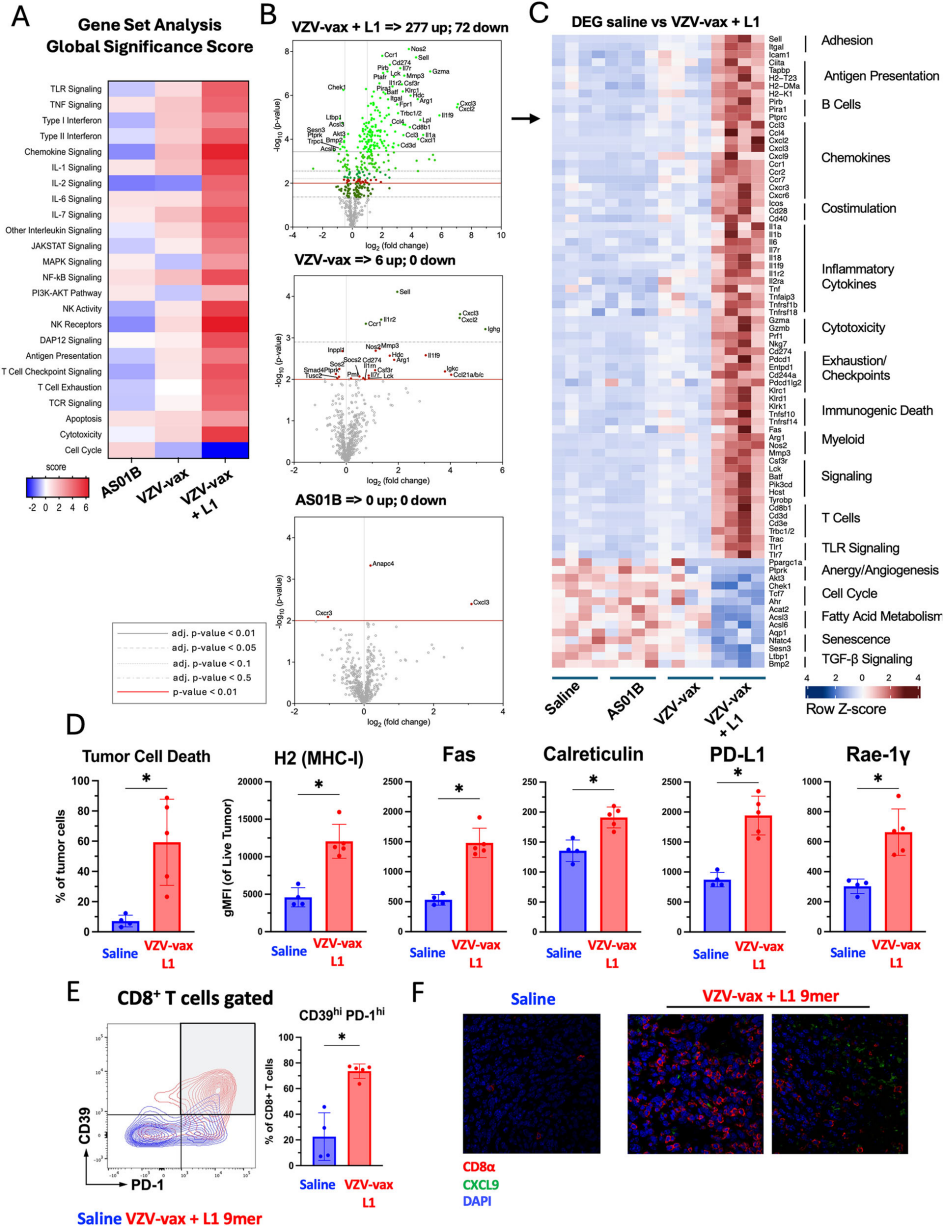
Tumor stress response and CD8^+^ T cell infiltration. Experimental design: C57BL/6 mice were prime-boost immunized intramuscularly with the VZV and HPV vaccines in opposite quadriceps prior to TC-1 tumor subcutaneous challenge one week after boost. When tumor reached a volume between 50 and 100 m^3^, mice were treated four times with saline, AS01B, VZV-vax alone (1/40^th^ of the human dose) alone or admixed with L1_165-173_ peptide (0.5 g). **A** Tumor RNA was extracted 24 h after the fourth injection and analyzed with the Nanostring Immune Exhaustion panel (*n* = 5). Heatmap shows the global significance score of each pathway measured in each group relative to saline-treated (**A**). **B** Volcano plot representation of the differential gene expression of each group compared with saline. Adjusted (adj.) *P* values were generated using the Benjamini–Yekutieli procedure and are shown as gray plain (*P* < 0.01), dashed gray (*P* < 0.05), dotted (*P* < 0.1) and dashdot (*P* < 0.5) lines. The plain red line indicates non-adjusted *P* value (*P* < 0.01). **C** The heatmap shows the Z-score of selected differentially expressed genes (DEG) selected from the saline-treated versus VZV-vax + L1_165-173_ peptide-treated groups. **D** The tumor cell stress response was measured by flow cytometry on CD45^-^ tumor cells. Data are shown as individual values and mean fluorescence intensity for each marker (MHC-I, Fas, calreticulin, PD-L1 and RAE-1γ) on live tumor cells with SE (*n* = 5 per group). **E** CD39 and PD1 expression by tumor infiltrating CD8^+^ T cells was measured by flow cytometry. Representative flow cytometry plot overlay showing CD39 and PD1 expression in saline (blue) and VZV-vax + L1_165-173_ peptide-treated (red) (*n* = 5). **D**, **E** Statistical analysis (*P < 0.05, n.s.: not significant) was performed using Dunn’s test for multiple comparison between groups (*n* = 5 per group). **F** Representative immunofluorescence image of tumor tissue sections from tumors treated with saline or VZV-vax + L1_165-173_ peptide. Sections were stained with CD8α (red) and CXCL9 (green) antibodies and nuclei were stained with DAPI (blue).

Next, we analyzed changes in gene expression using a mouse Immune Exhaustion panel (Nanostring). Global significant pathway score enrichment was established using saline-treated samples as baseline (Fig. 5A). Of note, TLR signaling, type 1 interferon signaling, chemokine signaling, antigen presentation, and T cell receptor signaling scores were slightly decreased in the group treated with AS01B alone, the VZV-vax adjuvant containing QS21 and MPLA. In contrast, these pathways were increased in VZV-vax-treated tumors and further increased by the addition of the HPV16 L1_165-173_ peptide (Fig. 5A). Interestingly, IT treatment with VZV-vax and VZV-vax with the HPV16 L1_165-173_ peptide induced a reduction in cell cycle scores, but not the treatment with the AS01B alone (Fig. 5A). Analysis of the differentially expressed genes (DEG) relative to the saline-treated group showed that AS01B did not induce significant changes at the level of individual gene expression (Fig. 5B). In contrast, VZV-vax IT injection alone induced the upregulation of L-selectin, CXCL3, CXCL2, CCR1, IL1R2 and immunoglobulin heavy constant gamma chain RNA (Fig. 5B). Other genes such as matrix metalloprotease 3, arginase 1 and granzyme A appeared upregulated but did not reach the significant threshold of the adjusted p-value (Fig. 5B). Strikingly, IT injection of VZV-vax admixed with the HPV16 L1_165-173_ peptide induced broad and significant changes in gene expression with 277 genes upregulated and 77 genes down regulated (Fig. 5B). Heatmap visualization of the differentially expressed genes between saline-treated and VZV-vax with L1_165-173_ peptide-treated groups showed an increase in genes involved in T cell adhesion (L-selectin, ICAM-1), antigen presentation (CIITA, TAP binding protein, H2-K), chemokines signaling (CXCL9, CXCL2, CXCL3, CCR1, CCR2, CXCR3), inflammation (IL-6, IL1, IL18, TNF), T cell signaling (Lck, Batf, CD3ε) and myeloid cells (Arginase 1, nitric oxide synthase 2), some of which were also increased in VZV-vax-treated groups, albeit to a lesser extent (Fig. 5C). Interestingly, genes associated with immunogenic cell death were also upregulated such as Fas, Tnfsf10 (TRAIL) and Tnfrsf14 (LIGHT). Finally, VZV-vax + HPV16 L1_165-173_ peptide intratumoral injection was associated with a decrease in expression of genes associated with the cell cycle (Chek1, aryl hydrocarbon receptor), fatty acid metabolism (acetyl coA transferase) and TGF-β signaling (latent transforming growth factor beta binding protein 1 and bone morphogenetic protein 2). Then, using flow cytometry, we assessed the expression of immunogenic cell death (Fas, calreticulin) and cellular stress (PD-L1, Rae-1γ) markers by tumor cells in tumor single cell suspensions (Fig. 5D). Intratumoral treatment with VZV-vax and HPV16 L1_165-173_ peptide led to increased cell death and increased expression of MHC-I, Fas, PD-L1, and Rae-1γ (Fig. 5D). Together, these results suggest that IT treatment with VZV-vax and HPV16 L1_165-173_ induces tumor cell death and increases tumor cells’ sensitivity to CTL recognition through an increase in MHC-I molecules, expression of death receptors (FAS), expression of NK receptor ligands (Rae-1-γ) and surface exposure of a marker of immunogenic cell death (Calreticulin). Next, we analyzed the tumor infiltration by CD8^+^ T cells using flow cytometry and confocal microscopy (Fig. 5E, F). VZV-vax and HPV16 L1 minimal peptide epitope IT treatment triggered the upregulation of CD39 and PD1 expression by tumor-infiltrating CD8^+^ T cells, suggesting local TCR-mediated activation of bystander anti vaccine CD8^+^ T cells^28^ (Fig. 5E). Increased infiltration was further confirmed by CD8 immunofluorescence staining which was associated with CXCL9 depot (Fig. 5F).

### Intratumoral VZV-vax and an HPV L1-derived MHC-I-restricted peptide induces potent tumor control enhanced by the addition of a peptide epitope derived from a tumor-specific antigen

**Fig. 6:**
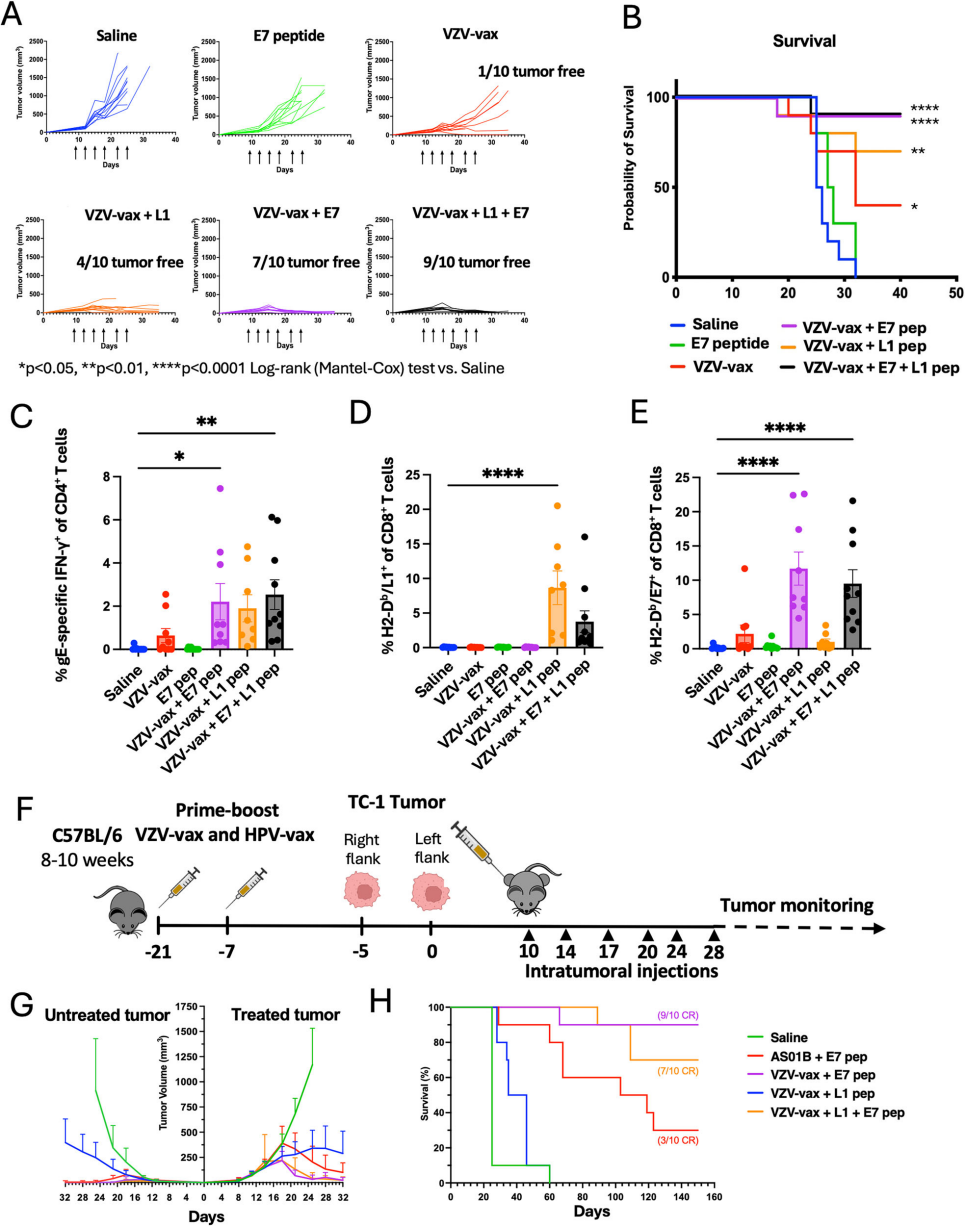
IT injection of combinations of VZV-vax and minimal epitopes derived from HPV L1 and/or E7 tumor-specific antigens elicits tumor control and potent abscopal antitumor responses. C57BL/6 mice were prime-boost immunized intramuscularly with the VZV and HPV vaccines in opposite quadriceps prior to TC-1 tumor subcutaneous challenge one week after boost. When tumor reached a volume between 50 and 100 m^3^, mice were treated six times with saline, E7_44-62_ peptide alone (2.5 μg), VZV-vax alone (1/40^th^ of the human dose) or admixed with the L1_165-173_ peptide (0.5 μg), E7_44-62_ peptide (2.5 μg), or a mixture of L1_165-173_ and E7_44-62_ peptides (0.5 μg and 2.5 μg, respectively). Mice were monitored twice a week for (**A**) tumor growth and (**B**) survival. **A** Tumor growth is shown as individual tumor volume for each group (*n* = 10 per group). **C** IFN-γ production was assessed after the IT treatment by intracellular staining of circulating CD4^+^ T cells after in vitro restimulation with VZV gE overlapping peptide library. Individual cytokine production is shown as individual values and mean percentage of CD44^+^CD4^+^T cells with SE (*n* = 10). Circulating (**D**) anti-HPV-vax L1-specific and (**E**) anti-tumor E7-specific CD8^+^ T cell responses were measured by dextramer staining after IT treatment. Data are shown as individual values and mean percentage of H2-D^b^/ L1_165-173_^+^ and H2-D^b^/E7_49-57_^+^ within CD44^+^CD8^+^ T cells. **F** Experimental design for a dual flank tumor challenge, C57BL/6 mice were immunized as previously described prior to TC-1 tumor subcutaneous challenge on the right and left flanks 5 days apart one week after boost. When primary tumors reached a volume between 50 and 100 m^3^, mice were treated six times with saline, AS01B and E7_44-62_ peptide, VZV-vax (1/40^th^ of the human dose) admixed with the L1_165-173_, E7_44-62_, or a mixture of L1_165-173_ and E7_44-62_ peptides. **B** Mice were monitored twice a week for (**G**) primary and secondary tumor growth and (**H**) survival. **A**, **G** Statistical analysis with Dunn’s test was performed for multiple comparisons of tumor growth between each group. **B**, **H** Survival comparisons between groups were assessed by Mantel–Cox test (****P < 0.0001, **P < 0.01, *P < 0.05, n.s. = not significant). **H** The number of complete responders are indicated in the survival plot (CR). **C**–**E** Statistical analysis with Dunn’s test was performed for multiple comparisons between groups of percent of gE-specific CD4^+^ T cells and L1- and E7-specific CD8^+^ T cells (****P < 0.0001, **P < 0.01, *P < 0.05, n.s. = not significant). **F** Tumor icon was created using Biorender.

Next, we investigated tumor control conferred by VZV-vax combined with the HPV16 L1_165-173_ minimal peptide epitope or a synthetic long peptide from the TC-1 tumor-specific antigen E7 (E7_44-62_) containing an MHC-I-restricted immunodominant epitope. C57BL/6 mice were immunized in a prime-boost regimen by concurrent intramuscular injection of the licensed VZV-vax and HPV-vax in opposite quadriceps. One week after the final immunization, mice were challenged subcutaneously with the TC-1 tumor cells. When tumors reached a volume between 50 and 100 mm^3^, we proceeded with six repeated injections of the tumor mass. Intratumoral injection of the tumor-specific E7_44-62_ peptide (2.5 μg) alone (without adjuvant) did not lead to a delay in tumor growth or increased survival (Fig. 6A, B). Intratumoral injection of the VZV-vax (equivalent to 1/40^th^ of the human dose) led to a delay in tumor growth compared to saline, and addition of the L1-derived peptide (0.5 μg) to VZV-vax caused tumor growth arrest with 4 out of 10 mice tumor-free (Fig. 6A, B). The combination of VZV-vax with the tumor-specific E7_44-62_ peptide led to tumor growth arrest with 7 out of 10 mice tumor-free and 2 out 10 mice with stable small tumors, and increased long-term survival (Fig. 6A, B). Finally, the IT injection of VZV-vax with the HPV16 L1_165-173_ peptide and the tumor-specific E7_44-62_ peptide led to tumor clearance of 9 out of 10 mice and long-term survival (Fig. 6A, B).

Next, we analyzed the anamnestic T cell responses after IT treatment. Intratumoral treatment with VZV-vax alone led to an increase in circulating IFN-γ-producing CD4^+^ T cells against the VZV gE protein as measured by ICCS after in vitro restimulation with the VZV gE OPL (Fig. 6C). The IT treatment with VZV-vax and HPV16 L1_165-173_ peptide led to an increase in IFN-γ-producing CD4^+^ T cells against the VZV gE protein (Fig. 6C), and to an increase in L1-specific CD8^+^ T cells measured by dextramer staining (Fig. 6D). The combination of VZV-vax with the tumor-specific E7_44-62_ peptide led to an increase in IFN-γ-producing CD4^+^ T cells against the VZV gE protein (Fig. 6C). While treatment with VZV-vax alone led to a modest increase in E7-specific CD8^+^ T cells measured by dextramer staining (Fig. 6E), these responses where further increased when VZV-vax was admixed with the tumor-specific E7_44-62_ peptide (Fig. 6E). Of note, we did not detect IFN-γ-producing CD4^+^ T cell responses against the E7_44-62_ peptide (Data not shown). The combination of VZV-vax with the HPV16 L1_165-173_ and the tumor-specific E7_44-62_ peptide led to an increase in IFN-γ-producing CD4^+^ T cells against the VZV gE protein (Fig. 6C) and of L1-specific and E7-specific CD8^+^ T cells (Fig. 6D, E) measured by dextramer staining. Interestingly, the percentage L1-specific CD8^+^ T cells was reduced in VZV-vax + L1_165-173_ + E7_44-62_ (mean=3.76±2.44) group compared to VZV-vax + L1_165-173_ alone (Mean=8.65±1.56) suggesting some MHC-I binding competition between these two H-2D^b^-restricted epitopes in favor of the tumor-specific epitope. Notably, IT injection of the E7_44-62_ peptide alone without adjuvant did not increase the frequency of E7-specific CD8^+^ T cells compared to saline-treated mice (Fig. 6E). Together, these data suggest that VZV-vax constitutes a potent platform for enhancing CD8^+^ T cells after IT delivery. These results suggest that a licensed VZV vaccine could be readily repurposed with other defined MHC-I-restricted peptide epitopes to harness preexisting CD8^+^ T cells against antiviral vaccines. Finally, these results demonstrate that the antitumor response could be further enhanced by incorporating tumor-specific antigens or neoepitopes.

We subsequently interrogated whether intratumoral injection of combinations of VZV-vax and viral vaccine-derived and/or tumor-specific antigens could elicit abscopal responses in a dual flank tumor challenge (Fig. 6F). The tumor challenge required an initial injection of TC-1 tumor cells to establish a primary tumor and a second injection on the opposite flank five days later, which allowed for a longer window of opportunity to treat and monitor tumor growth prior to euthanasia (Fig. 6G). Growth of the primary tumor after intratumoral injection of VZV-vax with the L1_165-173_ peptide was delayed, although not as pronounced as in other experiments using a single-flank tumor model (Fig. 6G). Interestingly, this group also showed a modest delay in the growth of the contralateral tumor (Fig. 6G). The combination of AS01B or VZV-vax with the E7_44-62_ peptide led to the control of the primary tumor and of the non-injected contralateral tumor (Fig. 6G). Interestingly, survival was increased and the growth of the primary tumor was delayed in the group treated with VZV-vax and the E7_44-62_ peptide compared to AS01B and the E7_44-62_ peptide (Fig. 6H). Finally, intratumoral injection of the combination of VZV-vax along with the E7_44-62_ peptide alone or admixed with the L1_165-173_ peptide led to the maximum tumor control of the primary and secondary tumor (Fig. 6G). Surprisingly, the abscopal tumor appeared to respond better than the treated primary tumor in groups including the E7 peptide, suggesting that larger, more established primary tumors may be more resistant to infiltrating CD8^+^ T cells.

### Harnessing CD8^+^ T cells induced by a SARS-CoV-2 mRNA vaccine confers tumor protection

**Fig. 7:**
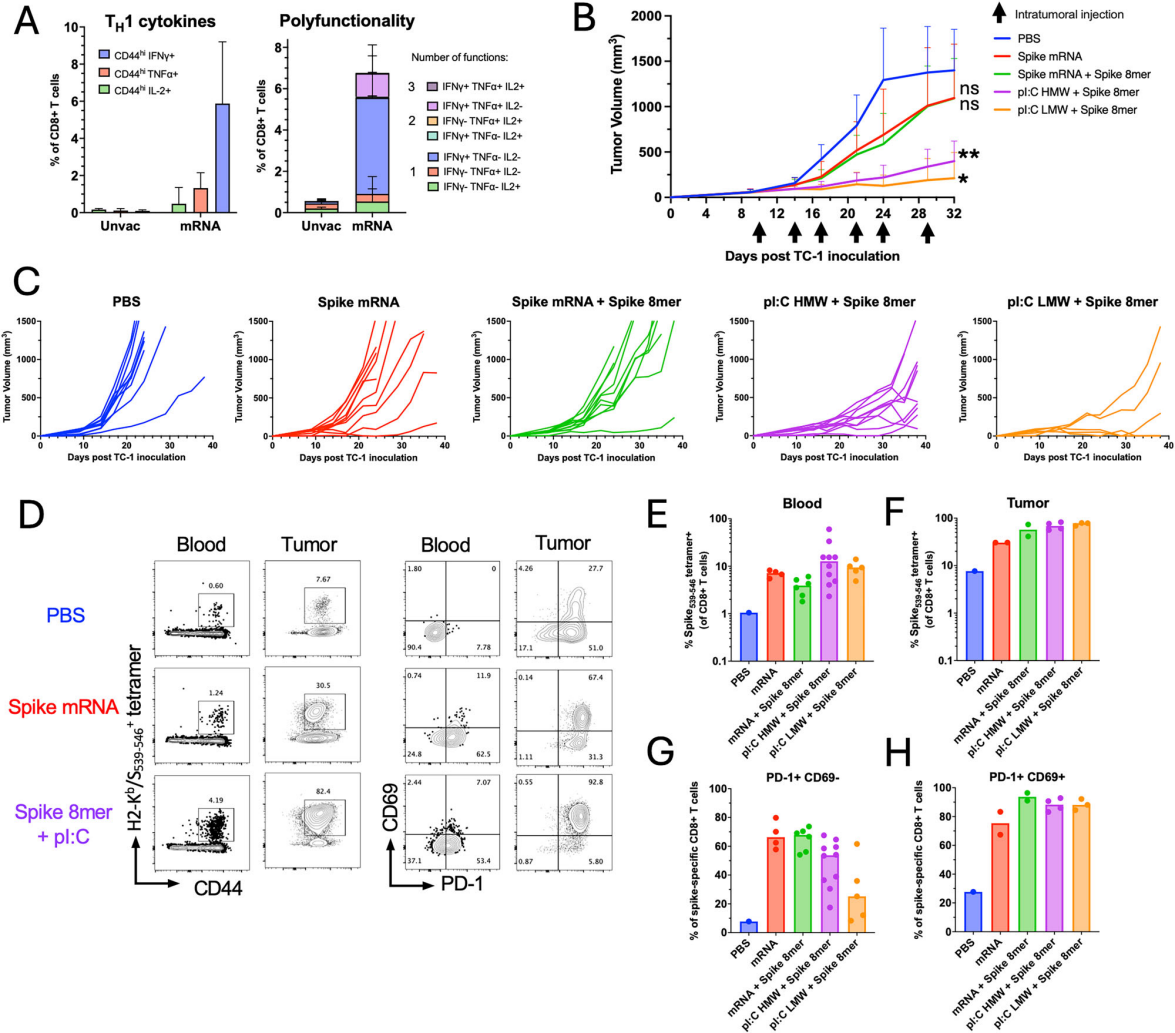
Harnessing a SARS-CoV2 mRNA vaccine confers tumor protection and elicits the recall of spike-specific CD8^+^ T cells in blood and tumors. C57BL/6 mice were prime-boost immunized intramuscularly with the SARS-CoV2 mRNA vaccine (1 μg mRNA) prior to TC-1 tumor subcutaneous challenge one week after boost. When tumor reached a volume between 50 and 100 m^3^, mice were treated six times with saline, SARS-CoV-2 mRNA vaccine (Spike mRNA) alone or admixed with the S_539-546_ peptide (Spike 8mer, 1 μg), or with polyI:C HMW or LMW (25 μg) admixed with the S_539-546_ peptide (1 μg). **A** TNF-α, IFN-γ and IL-2 production was assessed two weeks after prime-boost intramuscular immunization with Spike mRNA vaccine (*n* = 5 per group) or from naïve mice (*n* = 5 per group) by intracellular staining of circulating CD8^+^ T cells after in vitro restimulation with S_539-546_ peptide. Data are shown as individual values and mean single and multiple cytokine production percentage of CD44^+^CD8^+^ T cells with SE. **B** Tumor growth is shown as the mean with SE of tumor volume for each group injected intratumorally with saline, Spike mRNA, Spike mRNA with Spike 8mer or Spike 8mer with polyI:C (HMW or LMW). Statistical analysis (**P < 0.01, *P < 0.05, n.s.: not significant) was performed using Dunn’s test for multiple comparison between groups (*n* = 5 to 10 per group). **C** Spaghetti plots show the tumor growth for each individual mouse. Total H2-K^b^/S_539-546_ specific CD8^+^ T cells were quantified using MHC-tetramers in blood (**D**, **E**) and in tumors (**D**, **F**). PD1 and CD69 expression was assessed by antibody surface staining. **D** Representative FACS plot of H2-K^b^/S_539-546_-positive CD8^+^ T cells and, PD1 and CD69 expression by tetramer^+^CD8^+^ T cells. Data are shown as individual values and mean percentage of H2-K^b^/S_539-546_-positive in CD8^+^ T cells in blood (**E**) and in tumors (**F**) and the percentage of tetramer^+^CD8^+^ T cells PD1^+^CD69^-^ in blood (**G**) and PD1^+^CD69^+^ in tumors (**H**).

The COVID-19 pandemic led to an unprecedented mass scale vaccination campaign using novel mRNA platforms. The immunogenicity of the mRNA vaccines has been well documented, inducing broad immunity against the SARS-CoV-2 spike envelope antigen. Although protection is mostly mediated by antibodies against the spike protein, CD4^+^ and CD8^+^ T cell responses against spike epitopes occur even in older individuals^29^. Similarly, mRNA vaccines have been deployed to target cancer using personalized neoantigens and systemic vaccination^30^. Preclinical data suggest that such mRNA vaccines would also be amenable to IT delivery^31^. Here we sought to determine whether IT delivery of a licensed SARS-CoV-2 mRNA vaccine (Spikevax, Moderna) in pre-vaccinated mice could lead to tumor control (Fig. 7). In parallel, we investigated whether IT delivery of a spike-derived MHC-I-restricted epitope admixed with two forms of polyI:C, high and low molecular weight (HMW and LMW), could also lead to tumor control. First, we assessed the immune response induced by a prime-boost intramuscular vaccination regimen with the SARS-CoV-2 mRNA vaccine (equivalent to 1/50^th^ of the human dose) in C57BL/6 mice. We focused the analysis on CD8^+^ T cell responses directed against a previously reported immunodominant H2-K^b^-restricted epitope of the SARS-CoV-2 spike glycoprotein (S_539-546_)^32^. Two weeks after the last intramuscular immunization, blood leukocytes of SARS-CoV-2-vaccinated mice were restimulated in vitro with the S_539-546_ peptide and cytokine production was analyzed by intracellular staining (Fig. 7A). CD8^+^ T cells produced predominantly IFN-γ (mean=6% of CD8^+^ T cells) in response to S_539-546_ restimulation, to a lower extent TNF-α (mean=1.5% of CD8^+^ T cells) and a low level of IL-2 (mean=0.4% of CD8^+^ T cells). Overall, the majority of S_539-546_-specific CD8^+^ T cells were monofunctional, expressing IFN-γ, or TNF-α to a lesser extent (Fig. 7A).

C57BL/6 mice immunized with the SARS-CoV-2 mRNA vaccine were challenged with TC-1 tumor cells subcutaneously. Once the tumor reached a tumor volume between 50 and 100 mm^3^, mice were randomized according to tumor volume and treated IT with saline as a control, the SARS-CoV-2 mRNA vaccine (equivalent to 1/50^th^ of the human dose) alone, the SARS-CoV-2 mRNA vaccine with the S_539-546_ peptide (0.5 μg), or the S_539-546_ peptide (0.5 μg) admixed with polyI:C HMW or LMW (50 μg). IT injection of the SARS-CoV-2 mRNA vaccine induced a modest delay in tumor growth compared to the saline-treated group (Fig. 7B, C). The addition of the S_539-546_ peptide to the SARS-CoV-2 mRNA vaccine did not improve the delay of tumor growth observed with the SARS-CoV-2 mRNA vaccine alone (Fig. 7B, C). Notably, a combination of polyI:C HMW or LMW with the S_539-546_ peptide led to tumor control that was more pronounced than with the SARS-CoV-2 mRNA vaccine alone or with peptide (Fig. 7B, C).

Next, we analyzed the anamnestic response to IT delivery of mRNA or S_539-546_ peptide using a H2-Kb/S_539-546_ tetramer in the mice remaining at the end of the treatment. All treated groups displayed an enhanced spike-specific CD8^+^ T cell response compared with the saline-treated group in blood (Fig. 7D, E) and in tumors (Fig. 7D, F). We then analyzed the phenotype of H2-Kb/S_539-546_ tetramer-positive CD8^+^ T cells in blood and in tumors of the remaining mice. In blood, spike-specific CD8^+^ T cells in the saline-treated control group did not express PD-1 and CD69 (Fig. 7D, G). However, circulating spike-specific CD8^+^ T cells upregulated PD-1 after IT treatment with the mRNA vaccine or S_539-546_ peptide admixed with polyI:C HMW and LMW, suggesting that they were recently activated upon IT treatment. In the tumor, infiltrating spike-specific CD8^+^ T cells upregulated CD69 which is associated with tissue retention^33^ and PD-1 in groups treated IT with the spike mRNA vaccine or S_539-546_ peptide admixed with polyI:C as compared to the saline-treated control (Fig. 7D, H). These results indicate that IT injection with the SARS-CoV-2 mRNA vaccine or a spike-derived MHC-I-restricted minimal peptide epitope with adjuvant induces comparable CD8^+^ T cell anamnestic responses against the spike S_539-546_ epitope. However, substantial tumor suppressive responses were only observed in groups injected with the MHC-I-restricted minimal peptide epitopes with adjuvant, suggesting differential antigen processing and presentation in the antitumor response.

## Discussion

Harnessing preexisting antiviral immunity to treat cancer has been proposed in recent years^34^. Immunity against natural infection has been shown to induce durable functional T cell responses. However, the immunity status in the general population can vary widely in terms of exposure and duration since last exposure. In addition, the specificity and breadth of the T cell responses to virus-derived antigens in each patient is variable. Subunit or mRNA vaccines offer the potential to circumvent these two limitations to wide implementation. Firstly, shingles, HPV and SARS-CoV-2 vaccines are immunogenic in aged populations, in immunocompromised patients and in patients with cancer^12,30,35^. This implies that existing commercial vaccines could be effectively used in a priming phase prior to IT injection to boost and normalize the level of preexisting T cell responses. Second, compared to live virus vaccines, subunit vaccines contain a limited number of antigens which allows one to focus the design of IT delivered peptides to a narrower set of epitopes.

The optimal stage of implementation of IT delivery of anti-cancer agents is being debated as it was recently highlighted in the Society for Immunotherapy of Cancer (SITC) recommendations article on IT immunotherapy clinical trials^3^. The positive safety profile of antiviral vaccines in healthy populations suggests that this powerful approach could be applied across a broad range of tumors including premalignant tumors or in neoadjuvant settings. In addition, peptide cancer vaccines have a proven record of safety and tolerability. However, the identification of actionable tumor-specific antigens has remained elusive and requires a personalized identification of tumor epitopes^36^.

In this study, we show that preexisting T cell immunity against two common subunit vaccines and an mRNA vaccine could be leveraged for cancer immunotherapy. Specifically, we show that preexisting CD8^+^ T cell immunity against the HPV-vax and SARS-CoV-2 mRNA vaccine can be engaged upon IT administration of minimal MHC-I-restricted peptides but to a lesser extent by native vaccine antigens or whole vaccines. Others have shown that licensed vaccines can promote potent innate immune activation after IT injection leading to anti-tumor responses. These approaches demonstrate that the propensity of live-attenuated vaccines to trigger the TLR pathways, type 1 IFN response and dendritic cell activation is involved in the anti-tumor responses^5,6,37^. The mechanism of action of these approaches involves reprograming the suppressive myeloid compartment in the TME and promoting antigen presentation. In this study, we assessed two subunit vaccines and an mRNA vaccine with different formulations and innate stimulating properties and different outcomes in terms of tumor control. The HPV vaccine contains HPV VLP with exceptional innate immune stimulating properties^38^ and the ability to trigger MyD88 signaling and induce cytotoxic T lymphocyte (CTL) responses against the HPV16 L1 antigen. Surprisingly, our results clearly show that IT inoculation of the HPV-vax had no anti-tumor properties in the TC-1 model. The VZV-vax contains an adjuvant system composed of QS21 and MLPA incorporated in liposomes which can activate both the inflammasome and TLR4. The VZV-vax alone or in concert with the HPV L1 peptide induced strong antitumor responses and epitope spreading. In contrast, the SARS-CoV-2 mRNA vaccine is naturally immunogenic due to the properties of the ionized lipids and mRNA molecules that stimulate the inflammasome and MDA-5 but has limited TLR7/8 agonist activity compared to unmodified mRNA vaccines^17,39,40^. Our study shows that SARS-CoV-2 mRNA vaccine alone or in combination with a spike minimal peptide epitope conferred limited tumor control, compared to the same epitope combined with polyI:C.

PolyI:C, a synthetic double-stranded RNA molecules which targets the MDA-5/RIG-I pathway and TLR3 has been studied for many years as a standalone cancer therapy or vaccine adjuvant^23,41^. However, clinical benefits have been modest and combination with other immunotherapeutic agents might better harness the immunological properties of polyI:C and promote epitope spreading^42^. Our TME analysis showed that the engagement of adaptive T cell responses was required, generating a much stronger effect compared to the adjuvant component of the vaccines that we evaluated. Further studies are needed to assess the cooperation between innate components provided by the vaccine adjuvants and the quality of the T cell responses induced against the vaccine antigens. Indeed, it was shown recently that intratumoral polyI:C conditions the TME to potentiate CTL activity in solid tumors, which may limit suppression of the recruited CD8^+^ T cells^43^. Our tumor antigen agnostic combination approach which induces T-cell-mediated tumor cell killing and favor the release of tumor antigens in the presence of polyI:C appears well-suited to promote epitope spreading.

Other mechanisms, in addition to direct T cell cytotoxicity, could contribute to the anti-tumor responses observed in this study. It was recently shown that during cancer immunotherapy, T cell-mediated recruitment and activation of neutrophils was associated with bystander killing of tumor cells^44^. Our analysis of the TME shows that intratumoral T cell activation leads to neutrophil recruitment and upregulation of nitric oxide synthase 2 (Nos2) which could lead to tumor killing by bystander neutrophils. We also observed concomitant increases in IFN-γ and TNF-α in the TME which together could promote senescence of tumor cells as shown previously^45^. General tissue damage could also be invoked as peptide vaccine epitope presentation is not restricted to tumor cells. We observed a strong increase in cytotoxic genes such as granzyme A and genes associated with pyroptosis such as caspase-1^46^. Together our data suggest that some or all these mechanisms could be involved together in the tumor clearance observed after intratumoral reactivation of vaccine-specific T cells.

Our results show that repeated IT treatment with HPV-vax did not boost the CD8^+^ T cell response against L1 antigen, which is in agreement with a previous report showing that anti-VLP antibodies interfere with the recall of CD8^+^ T cells against linked heterologous antigens^47^. The advantage of the injection of peptides over VLPs was evident as peptides may be able to bypass the diminished cross-presentation of HPV VLP vaccine-derived epitopes due to preexisting anti-VLP vaccine antibodies^47^. Indeed, our results show that minimal peptide epitopes are extremely potent at inducing CTL killing and amplifying CD8^+^ T cells both in the tumor and in the circulation, yet they do not boost preexisting antibody responses, likely due to their low molecular weight. Using an alternative approach, it was recently shown that pre-vaccination of mice with a reovirus-derived minimal peptide epitope promoted anamnestic T cell responses against an oncolytic reovirus without inducing interfering antibodies to the injected reovirus, leading to enhanced therapeutic responses^48^.

In contrast to interference with the recall of preexisting CD8^+^ T cells, VZV-vax-specific CD4^+^ T cells did not appear to be impeded by preexisting antibodies, as IT injection of the gE-based VZV vaccine led to efficient recall of CD4^+^ T cell responses and at least partial tumor control. We speculate that the presentation of protein antigens in the MHC-II presentation pathway is not affected or may be increased in the presence of preexisting antibodies. Also, scavenging of soluble versus alum-adsorbed VLP antigens as antibody complexes may differentially impact antigen cross-presentation of this antigen to T cells. This led us to assess a combination of VZV-vax with a minimal MHC-I-restricted epitope which could avoid the need of selecting MHC-II-restricted epitopes, thus reducing the peptide selection to a set of MHC-I restricted peptides and thereby allowing the implementation of this approach in a genetically diverse population. The ability of VZV-vax to enhance CD8^+^ T cell responses against a co-delivered tumor-specific antigen was surprising as the anti-gE response after VZV-vax is strongly biased toward CD4^+^ Th1 cells and antibodies. It was previously shown that type-2 conventional dendritic cells, which are typically associated with the induction of CD4^+^ T cell responses, are activated by the adjuvant AS01B but could also prime CD8^+^ T cells against an OVA-derived MHC-I-restricted model epitope after intramuscular immunization^49^. Our results expand these findings and show that VZV-vax induces cytotoxic and pro-immunogenic changes in the TME and can be combined with vaccine- or tumor-derived MHC-I-restricted peptide epitopes in the context of IT therapy and vaccination.

We reasoned that mRNA vaccines could be an excellent candidate to harness preexisting vaccine T cell immunity. First, antigen delivery to the MHC-I pathway after translation of intracellular mRNA can be achieved by direct presentation as well as cross-presentation of myocyte-derived antigens^50–52^. Second, lipid nanoparticles encapsulating the mRNA encoding a viral antigen do not induce vehicle-inactivating antibodies thereby allowing for repeated administration. Our analysis of anamnestic CD8^+^ T cell responses against a spike epitope shows that, indeed, IT delivery of an mRNA vaccine led to the systemic and local activation of CD8^+^ T cells. However, this expansion did not lead to strong anti-tumor effects in comparison to intratumoral delivery of an MHC-I-restricted spike-derived peptide. This suggests that the mRNA IT treatment could induce lower levels of cell surface MHC/peptide complexes compared to direct loading of MHC-I molecules by exogenously delivered minimal peptides, or that fewer tumor or non-tumor cells are transduced by the mRNA vaccine, thereby limiting antigen presentation to incoming CD8^+^ T cells and reducing local immune activation and cytotoxicity. It is noteworthy that the intratumoral injection of the spike-derived MHC-I-restricted epitope to mRNA-vaccinated mice rarely induced complete tumor regression, in contrast to delivery of the HPV L1-derived MHC-I-restricted epitope, or even delivery of the VZV gE-derived MHC-II-restricted epitope, in correspondingly pre-vaccinated mice. Whether this difference in potency reflects functional differences in the characteristics of the T cells induced by the vaccines remains to be determined. Supporting this conjecture, we have shown previously that IT injection of MHC-I-restricted peptides recognized by memory MCMV-specific CD8^+^ T cells was more effective in control of solid tumors compared to peptides recognized by their more abundant terminally differentiated, inflationary counterparts^19^.

Repurposing drugs for cancer therapy has been an intense area of investigation. Recently, the AS01B adjuvant was investigated for IT delivery in refractory melanoma in combination with immune checkpoint inhibitors^53^. Intralesional injection of HPV-vax to treat a cutaneous basaloid squamous cell carcinomas was reported in a case study but the contribution of preexisting immunity was not assessed^54^. However, the opportunity of taking advantage of vaccine immunogenicity in randomized clinical trials remains to be established. In addition, intratumoral delivery might require vaccine reformulation to maximize adjuvant and antigen concentration and to achieve a smaller volume amenable to IT delivery.

We believe that this approach to cancer immunotherapy has several attractive features particularly with respect to potential applications in low-resource settings. It could potentially generate a simple off-the-shelf minimal peptide-based product, coupled with widely administered licensed vaccines, that would be applicable across a broad spectrum of cancer types without the need for sophisticated molecular profiling or development of a companion diagnostic. It acts both as a rapid cytotoxic agent and as an antigen-agnostic vaccine, and it is notably effective at inducing tumor clearance (in our model) without immune checkpoint blockade. Lastly, it is a biological immunotherapy with the potential for unlimited treatments because the minimal peptide epitopes are unlikely to induce inactivating antibodies, in contrast to oncolytic virus-based therapies.

We employed an HPV16 E7 peptide as our model tumor antigen because the oncoproteins E6 and E7 are exceptionally attractive tumor-specific targets. They are selectively retained and expressed in over half of the more than 600,000 annual global cases of cervical cancer, most of which occur in low-resource settings, and in approximately 90% of HPV-associated vulvar, vaginal, anal and oropharyngeal cancers^55–57^. In addition, E6 and E7 are only approximately 158 and 98 amino acids in length, respectively, making overlapping peptide libraries to cover the diversity of MHC-I alleles in the human population a practical consideration for a widely applicable off-the-shelf HPV-associated cancer therapy. While most peptide-based vaccination approaches are based on systemic delivery^58^, a preclinical study investigating the IT delivery of an HPV-derived tumor-associated peptide with adjuvant showed improved tumor control^59^. Our results indicate that the tumor nodules, when injected with a VZV-vax, are a bona fide immunization site for tumor-specific peptide epitopes which can increase the epitope spreading observed in a tumor-antigen approach, therefore leading to potent abscopal responses.

Limitation of the study: The evaluation of our approach relies on a fast-growing syngeneic tumor model. These models do not recapitulate the slow process of cancer progression from initiation to premalignant, invasive, and metastatic stages. While these findings in syngeneic models are encouraging, it is unclear whether this strategy will be as effective in genetically diverse, spontaneously arising tumors in older individuals that have evolved over extended periods to escape the host’s antitumor immunity. Currently, a National Cancer Institute-sponsored clinical trial in companion dogs is underway to assess this IT approach in spontaneous tumors. Such trials are needed to provide invaluable information on schedule, dosage, efficacy and tolerability of IT injection of anti-viral vaccines and vaccine-derived peptides as a prelude to human trials.

## Methods

### Cell lines and vaccines and in vivo reagents

TC-1 cells were obtained from Dr Tzyy-Choou Wu (The Johns Hopkins University) and maintained in Roswell Park Memorial Institute Medium (RPMI) 1640 (Gibco) supplemented with 10% fetal bovine serum (FBS, SIGMA), L-Glutamine (Gibco) and 0.4 mg/mL G418 (Invivogen)^20^. The commercial HPV vaccine (Gardasil-9, Merck), VZV vaccine (Shingrix, GSK) and SARS-CoV-2 mRNA vaccine (Spikevax, Moderna) were obtained from the division of veterinary resources (DVR) of the NIH. Minimal peptide epitopes (>90% purity) derived from HPV16 L1 capsid protein (L1_165-173_, AGVDNRECI), VZV glycoprotein E (gE_71-90_, SRKAYDHNSPYIWPRNDYDG), SARS-CoV-2 spike envelope (S_539-546,_ VNFNFNGL) and HPV16 E7 oncoprotein (E7_44-62_, QAEPDRAHYNIVTFCCKCD) were obtained from Genscript. Lyophilized peptides were resuspended in dimethylsulfoxide (DMSO) or water and stored at -80 °C for long-term and -20 °C for short-term storage according to manufacturer’s guidelines. Lyophilized high and low molecular weight (HMW and LMW, respectively) polyI:C were obtained from Invivogen and prepared in saline following manufacturer’s guidelines.

### Mice and in vivo experimental procedures

C57BL/6 female mice were purchased from the Jackson Laboratory and maintained under specific pathogen-free conditions. All animal experiments followed approved protocols by the National Cancer Institute Animal Care and Use Committee. All injections (subcutaneous, intramuscular, and intratumoral) and blood sampling were performed on anesthetized animals. Anesthesia was induced by inhalation with 4% isoflurane in oxygen and maintained with 2% isoflurane in oxygen. Euthanasia was performed on isoflurane-anesthetized mice by cervical dislocation.

Mice 8-10 weeks old were vaccinated by the intramuscular route following a prime-boost regimen two weeks apart. For dual vaccination with VZV-vax and HPV-vax, each vaccine was injected by the intramuscular route in opposite quadriceps.

Mice vaccinated with VZV-vax and HPV-vax received a dose equivalent to 1/10^th^ of the recommended human dose corresponding to 50 μl of each vaccine suspension. Mice vaccinated with SARS-CoV-2-vax received a dose of 1/50^th^ of the recommended human dose corresponding to 10 μL of the mRNA-lipid nanoparticle suspension completed to a final volume of 50 μL with sterile PBS immediately before injection.

For tumor challenge, mice were injected subcutaneously on the flank with 5×10^5 TC-1 tumor cells one week after the booster dose. When tumors reached a volume between 50 and 100 mm^3^, mice were randomized into treatment groups according to tumor size and the following day, treated intratumorally twice a week for 6 consecutive IT injections for survival experiments and 4 times for TME analysis. Tumor growth was monitored twice a week using a digital caliper (Mitutoyo) until tumors reached a volume of 1500 mm^3^ or humane endpoint. In some experiments, CD8^+^ T cells were depleted by antibody during IT treatment. Mice were injected with 200 μg of an anti-mouse CD8 antibody (clone 2.43, BioXcell InVivoPlus) or a rat IgG2b control antibody (clone LTF-2, BioXcell InVivoPlus) diluted in 200 μl phosphate-buffered saline (PBS) on day 3 and 1 prior to the first IT injection and on day 1 prior to each subsequent IT injection.

### Blood and tissue collection

Blood samples were collected in ethylenediaminetetraacetic acid (EDTA)-coated tubes (SAI Infusion Technologies) by retroorbital method on isoflurane-anesthetized mice at the time point specified in the figure legends. Plasma was collected by centrifugation at 5000 rpm, and red blood cells were lysed in ammonium-chloride-potassium (ACK) buffer (Life technologies). Spleen and tumors were dissected on euthanized mice and finely minced prior to incubation at 250 rpm at 37 °C in RPMI supplemented with 2% fetal bovine serum (FBS), 0.5 mg/mL Collagenase A (Roche) and 0.1 mg/mL DNase1 (Roche) for 15 min (spleen) and 30 min (tumor). Single-cell suspensions were passed through a 70-μm filter, and cells were counted for immediate flow cytometry analysis or peptide restimulation. Alternatively, some tumor tissues were snap frozen in liquid nitrogen and stored at -80C until processing for RNA and protein extraction or embedded in Optimal Cutting Temperature (O.C.T.; Tissue-Tek) and stored at -80 °C prior to microscopy analysis.

### Enzyme-linked immunosorbent assay (ELISA) for anti-gE and anti-L1 IgG titers

Plasma samples were assayed by ELISA for antigen-specific IgG response. Briefly, high-binding plastic 96-well plates (Immunlon 4HBX) were coated with either HPV16 L1 VLP or recombinant gE (Shingrix) at 100 ng per well in PBS. Plates were washed in PBS supplemented with 0,05% Tween20 (PBST) and blocked with PBS supplemented with 0.5% dry skim milk and 0.1% FBS for 2 h at room temperature (RT). After 2 washes in PBST buffer, serial dilutions of the samples in PBS supplemented with 0.5% dry skim milk were added to the plate and incubated for 2 h at RT. After 3 washes in PBST buffer, a horse radish peroxidase (HRP)-conjugate goat anti-mouse IgG antibody (Southern biotechnology) was added to the plate for 1 h at RT. After 3 washes, plates were developed in 3,3′,5,5′-Tetramethylbenzidine (TMB, KPL) for 10 min and the colorimetric reaction was stopped by adding 1 volume of HCl 1 N. Absorbance was read on a spectrophotometer at 450 nm and a reference filter at 540 nm. End point titers were interpolated using Prism (Graphpad).

### Flow cytometry

All stainings were performed in PBS supplemented with 2% FBS and 2 mM EDTA (FACS buffer). Prior to staining with antibodies, single-cell suspensions were incubated FACS buffer containing an anti CD16/32 antibody (2.4G2; BioXCell) to block Fc receptors. After antibody staining, samples were incubated with amine-reactive dyes to discriminate dead cells (Invitrogen LIVE/DEAD Fixable Yellow or Lime Dead Cell Stain Kit), washed twice in FACS buffer, and incubated in fixation buffer containing paraformaldehyde (PFA) (Biolegend) prior to acquisition on a flow cytometry analyzer instrument. Single-color compensation controls and fluorescence minus one (FMO) controls were included in each staining. Samples were acquired with a High Throughput Sampler (BD Biosciences) and analyzed using FlowJo v10 (TreeStar).

#### Antigen-specific CD8^+^ T cell staining

Single cell suspensions were incubated in FACS buffer containing an anti CD16/32 antibody (24G2; Bio X Cell) with APC-conjugated H2-D^b^/E7_49-57_, H2-D^b^/L1_165-173_ dextramers (Immudex) or a PE-conjugated H2-K^b^/S_539-546_ tetramer (NIH Tetramer Facility) for 30 min at RT, followed by an additional incubation of 30 min at 4 °C with anti-CD45-BV421, CD4-APC/Cy7, CD8α-BV570, CD44-Percp/Cy5.5, PD-1-FITC and CD69-PE/Cy7 antibodies (Supplementary Tables 1 and 2) for analysis on a BD FACS Canto II instrument or anti-CD39-BUV395, CD11b-BUV496, NK1.1-BUV737, CD3-BV421, CD8a-BV510, CD45-BV605, PD-1-BV711, CXCR3-BV786, CD62L-FITC, CD44-PerCP/Cy5.5, CD127-PE, CD69-PE/Cy7 and CD4-APC/Cy7 antibodies (Supplementary Table 3) for analysis on a BD Fortessa instrument.

#### Tumor myeloid infiltrate staining

After Fc receptor blocking, single cell suspensions were stained with anti-CD45-BV421, Ly6G-APC/Cy7, CD11c-PE, Ly6C-PE/Cy7, CD11b-FITC, IAIE-PerCP/Cy5.5, F4/80-APC and CD19-BV570 antibodies (Supplementary Table 4) for 30 min at 4 °C. Samples were acquired on a BD FACS CANTO II instrument.

#### Tumor cells phenotyping

After Fc receptor blocking, single cell suspensions were stained with anti-CD103-BUV496, CD11b-BUV395, CD45-BV605, PD-L1-BV421, Fas-PerCP-Cy5.5, H-2-FITC, Thy1.1-PE/Cy7, Rae1-γ-PE, H-2Db-APC/Fire-750 and calreticulin-APC antibodies (Supplementary Table 5) for 30 min at 4 °C. Samples were acquired on a BD Fortessa instrument.

#### Intracellular cytokine staining (ICCS)

In vitro antigen-specific T cell stimulation was performed with the indicated VZV gE and HPV L1 overlapping peptide libraries (OPL) (Pepmix, JPT technology) or minimal peptide epitopes HPV L1_165-173_, VZV gE_71-90_ and SARS-CoV-2 S_539-546_ (Genscript) at a concentration of 0.5 μg /mL for OPL (15mers, offset of 4 residues) and 5 μg/mL for minimal peptides. Splenocytes were incubated for 6 to 12 h at 37 C°C in 5% CO2 in RPMI 1640 supplemented with 10% FBS, L- Glutamine, 2-mercaptoethanol, sodium pyruvate, antibiotics, and brefeldin A and monensin (BD Bioscience). Negative and positive controls were obtained after restimulation with medium only, or phorbol 12-myristate 13-acetate (PMA)/ionomycin (Biolegend), respectively. After incubation, cells were washed and labeled with LIVE/DEAD Fixable Yellow Dead Cell Stain Kit (Invitrogen) followed by surface staining with CD3-BV421, CD4-APC/Cy7, CD8α-PE and CD44-PE/Cy7 antibodies (Supplementary Table 6). Cells were then fixed and permeabilized using commercial buffers (Biolegend). Intracellular staining of cytokines was performed in saponin containing permeabilization buffer (Biolegend) with anti IFN-γ-FITC, TNF-α-APC and IL-2-PerCP/Cy5.5 or isotype control antibodies (Supplementary Table 6). Samples were acquired on a BD FACS Canto II instrument.

### Multiplex cytokine and chemokine quantification

Cytokines and chemokines were quantified using the Cytokine Release Syndrome multiplex panel containing bead/antibody pairs for CCL2, IL-10, CCL4, IFN-α, CXCL9, CXCL10, TNF-α, IL-6, VEGF, IL-4, CCL3, IFN-γ, and GM-CSF (Legendplex, Biolegend) following manufacturer’s instructions. Tumor samples were obtained 36 h after the last IT treatment and immediately frozen in liquid nitrogen as indicated in the figure legend. Tissue lysates were obtained by bead bashing using a TissueLyser LT (Qiagen) in PBS, supplemented with 2 mM Mg2^+^, 25 U/ml Benzonase (Sigma) and a protease inhibitor cocktail (Complete mini, Roche). Protein content was assessed using a bicinchoninic acid (BCA) assay kit (Pierce) to normalize the amount of protein used in each test (10 μg to 50 μg).

### Confocal microscopy analysis

Fresh tumor tissues were fixed in 4% PFA for 2 h at RT and incubated in 15% sucrose overnight at 4 °C and in 30% sucrose for 24 h at 4 °C prior to embedding and freezing in O.C.T (Tissue-Tek) in liquid nitrogen vapor. Tissue blocks were stored frozen at -80 °C. Six-micron tissue sections were cut with a cryotome and fixed in cold ethanol for 10 min. Tissue sections were then stained with primary anti-CXCL9 and Alexa-594 anti-CD8α antibodies followed by incubation with an anti-Armenian Hamster-Alexa-488 secondary antibody (Biolegend) and tissue sections were then labeled with 4,6-diamidino-2-phenylindole (DAPI) for nuclei staining and mounted with an antifade reagent prior to addition of coverslips (Molecular Probes). Confocal images were acquired at the Confocal Microscopy Core Facility, Center for Cancer Research, NCI, NIH, with Zeiss ZEN software on a Zeiss LSM 780 Confocal system using a 40X oil immersion objective and 364-nm, 488-nm, and 543-nm lasers. Images were analyzed using Image J, and color channel levels were adjusted uniformly across images.

### Gene expression analysis in tumor tissues

Tumor tissues were collected 48 h after the last IT injection and flash-frozen in liquid nitrogen. Frozen tumors were placed in a 2 mL tube with the Ceramic Beads Precellys Kit (Bertin instruments) filled with 1 mL Trizol (Thermo Fisher) and processed in a Precellys24 Homogenizer (Bertin Instruments). A volume of 0.2 mL of chloroform was added to the tumor lysate and mixed thoroughly before centrifugation at 12,000 rpm for 15 min at 4 °C. The aqueous phase was further processed using the RNeasy Mini QIAcube Kit (Qiagen). Purified RNA was quantified using a Nanodrop device (Nanodrop Products) and quality control was performed with a TapeStation instrument (Agilent). A total of 200 ng of RNA was used for gene expression analysis using the mouse nCounter Immune Exhaustion Profiling Panel (Nanostring Technologies). Sample preparation and hybridization was done at the Center for Cancer Research Genomic Core Facility following the manufacturer’s instruction. The gene expression data were normalized using the nSolver Analysis Software 4.0 (Nanostring Technologies). Further bioinformatic analysis and data visualization were performed using the nCounter Advanced analysis software and R packages ggplot2, pheatmap and complexheatmap.

### Statistics

Statistical analyses were performed using Prism software (GraphPad Software, Inc.). Unpaired Mann–Whitney tests were used to analyze statistical differences between two groups. Dunnet’s test was used for multiple comparisons to a single control group. The Mantel–Cox test was used for survival analysis. For volcano plots, a threshold of log fold-change 2 and 0.5 and a p-value of 0.05 were used to define significant changes gene expression. Statistical details are indicated in the figure legend.

## Supplementary information

Supplementary information file

## Data Availability

The data that support the findings of this study are included in the manuscript. All materials used in this manuscript are available from the corresponding author upon reasonable request. Nanostring data have been deposited in the Gene Expression Omnibus (GEO) and is accessible through GEO series accession number GSE290583.
