# Supplementary material for: Repurposing anti-viral subunit and mRNA vaccines T cell immunity for intratumoral immunotherapy against solid tumors
Source: NPJ Vaccines. 2025 Apr 25;10:84. doi: 10.1038/s41541-025-01131-y (PMC12032097; doi:10.1038/s41541-025-01131-y)
Supplement: Supplementary file 1 — Supplementary information file [file 41541_2025_1131_MOESM1_ESM.pdf]

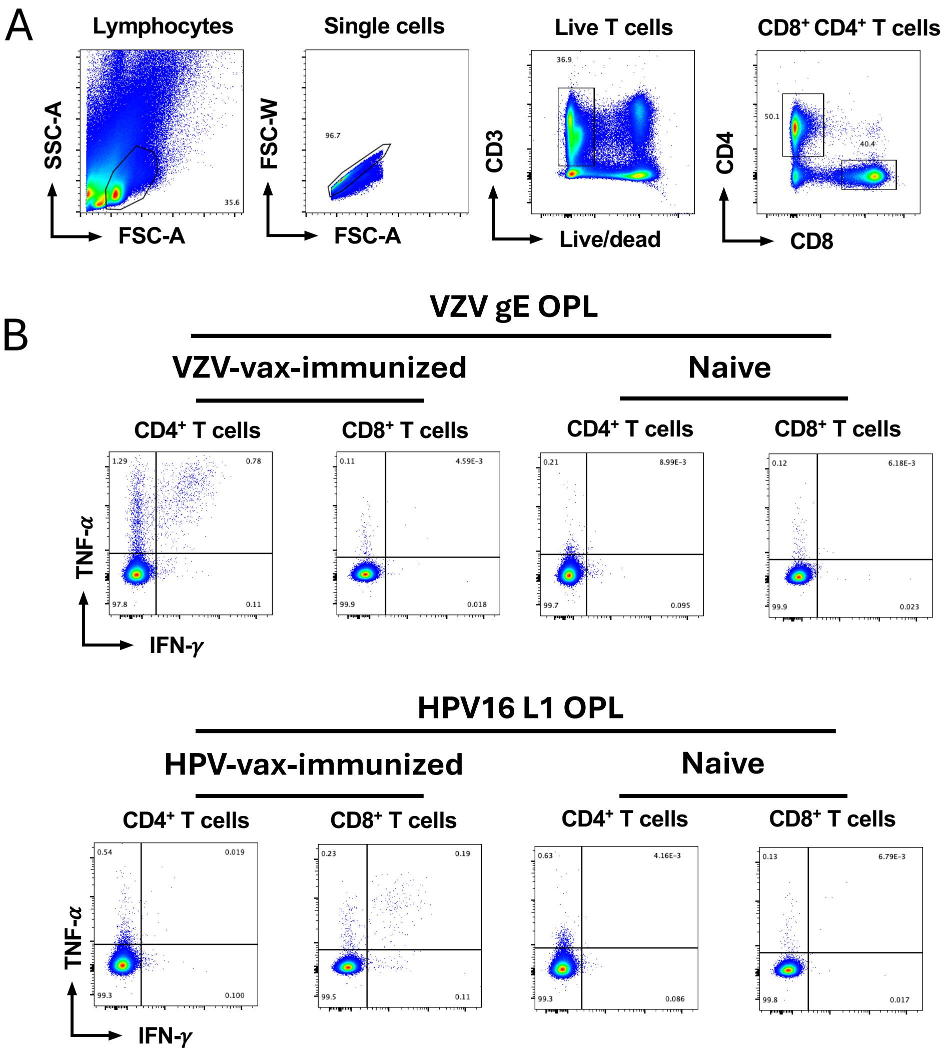

Supplementary Figure 1: Flow cytometry gating strategy for the analysis of IFN-γ and TNF-α CD4⁺ and CD8⁺ T cells. Analysis of IFN-γ and TNF-α production by spleen CD4⁺ and CD8⁺ T cells after in vitro stimulation with overlapping peptide libraries derived from VZV gE and HPV L1 antigens or medium only. (A) Flow cytometry gating strategy of CD8+ T cells for ICCS. (B) ICCS staining IFN-γ and TNF-α by CD4⁺ and CD8⁺ T cells in VAZ-vax- and HPV-vax-immunized, or naïve mice. Each FACS plot represents a concatenate in each experimental condition.

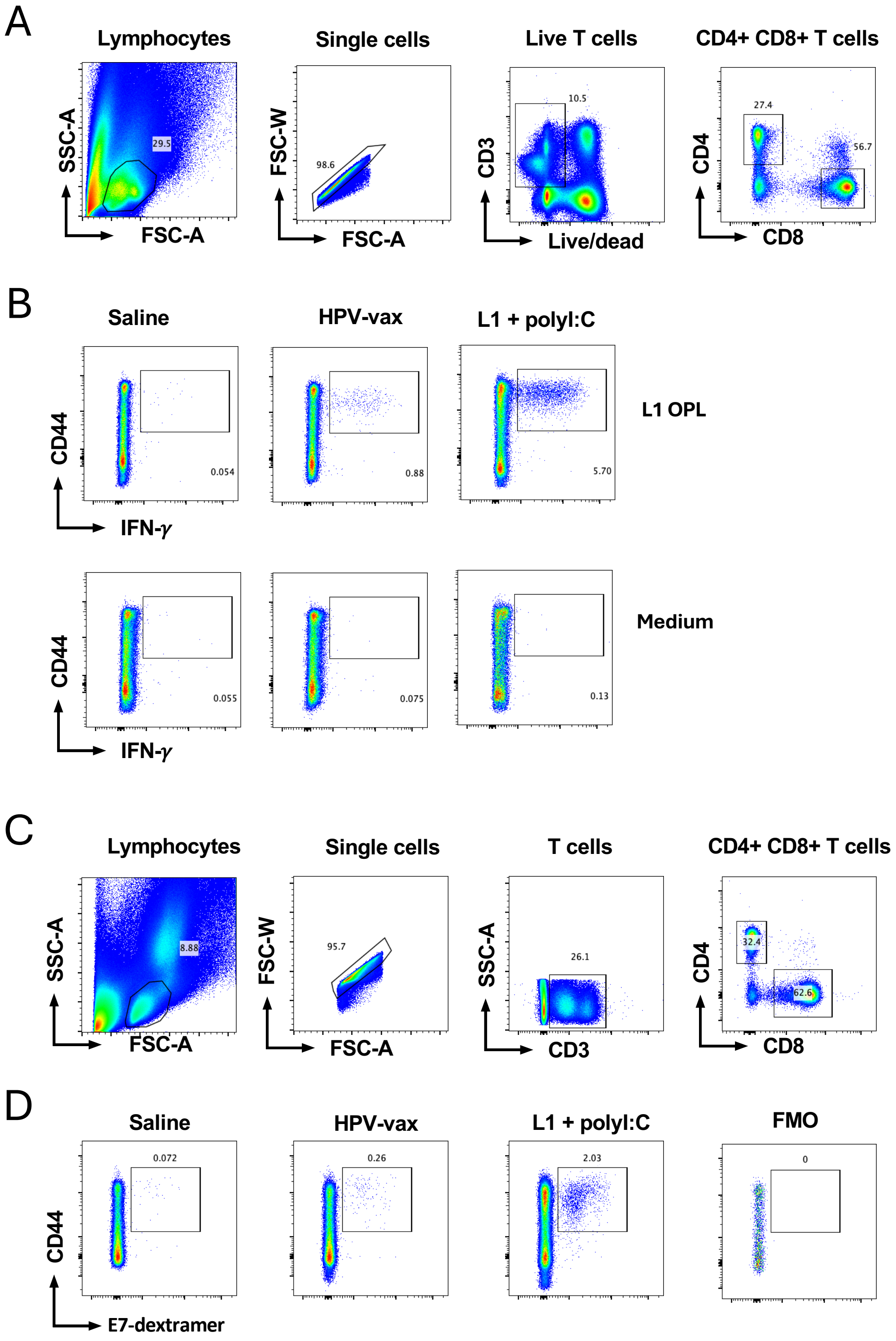

Supplementary Figure 2: Flow cytometry gating strategy for the analysis of IFN-γ-producing L1-specific CD8^{+} T cells and E7-specific CD8+ T cells. (A) Gating strategy of CD8+ T cells for ICCS. (B) ICCS staining of IFN-γ and TNF-α by CD4^{+} and CD8^{+} T cells after IT treatment with saline, HPV-vax or L1 polyI:C. (C) Gating strategy of CD8+ T cells for dextramer staining. (D) Dextramer staining of CD8+ T cells after IT treatment with saline, HPV-vax or L1 polyI:C. Each FACS plot represents a concatenate in each experimental condition.

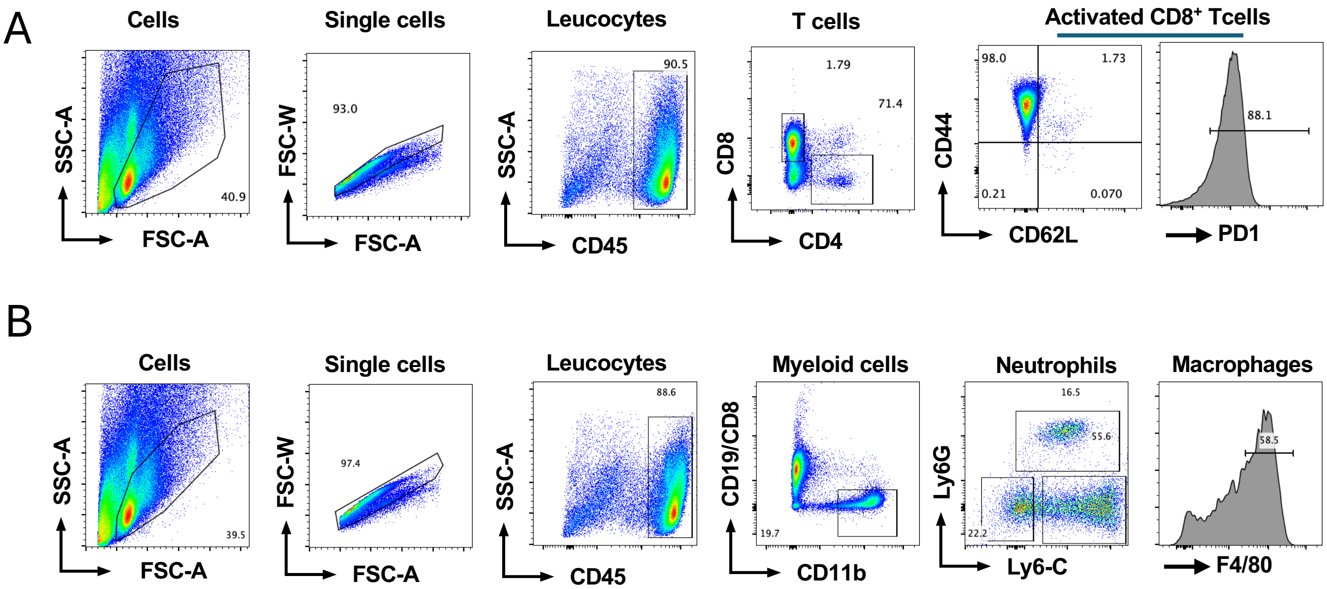

Supplementary Figure 3: Flow cytometry gating strategy for the analysis the myeloid tumor infiltrate. (A) Gating strategy of activated CD8^{+} T cells defined as CD45^{+}CDCD4^{-} CD8^{+}CD44^{+}CD62L^{-}PD1^{+}. **(B**) Gating strategy of Neutrophils defined as CD45^{+}CD19^{-}CD8^{-} CD11b^{+}Ly6G^{+}Ly6C^{-}, Monocytes defined as CD45^{+}CD19^{-}CD8^{-}CD11b^{+}Ly6G^{-}Ly6C^{+} and macrophages defined as CD45^{+}CD19^{-}CD8^{-}CD11b^{+}Ly6G^{-}Ly6C^{-}F480^{+}.

Supplementary Table 1. List of MHC-I multimers reagent for antigen-specific CD8+ T cells staining analyzed on BD FACS Canto II instrument

Antigen	Clone	Fluorochrome	Company	Catalog#
**$H2\text{-}D^b/E7_{49\text{-}57}$**	N/a	PE	Immudex	JA02195PE
**$H2\text{-}D^b/L1_{165\text{-}173}$**	N/a	APC	Immudex	JA03599APC
**$H2\text{-}K^b/S_{539\text{-}546}$**	N/a	APC	NIH tetramer	N/a

Supplementary Table 2. List of antibodies and MHC-I multimers used for antigen-specific CD8+ T cells staining analyzed on BD FACS Canto II instrument

Antigen	Clone	Fluorochrome	Company	Catalog#
**CD3**	17A2	BV421	Biolegend	100228
**CD4**	RM4-5	APC/Cy7	Biolegend	100526
**CD8**	53-6.7	BV570	Biolegend	100740
**MHC-I multimer**		APC		
**CD44**	IM7	PERCP/Cy5.5	Biolegend	103032
**CD69**	H1.2F3	PE/Cy7	Biolegend	104512
**MHC-I multimer**		PE		
**PD1**	29F.1A12	FITC	Biolegend	135214

Supplementary Table 3. List of antibodies and MHC-I multimers used for antigen-specific CD8+ T cells staining analyzed on BD Fortessa instrument

Antigen	Clone	Fluorochrome	Company	Catalog#
**CD39**	Y23-1185	BUV395	BD Horizon™	567264
**CD11b**	M1/70	BUV496	BD OptiBuild™	749864
**NK1.1**	PK136	BUV737	BD OptiBuild™	741715
**CD3**	17A2	BV421	BioLegend	100228
**CD8a**	53-6.7	BV510	BioLegend	100752
**CD45**	30-F11	BV605	BD Horizon™	563053
**PD-1**	29F.1A12	BV711	BioLegend	135231
**CXCR3**	CXCR3-173	BV786	BD OptiBuild™	741032
**CD62L**	MEL-14	FITC	BioLegend	104406
**CD44**	IM7	PerCP-Cy5.5	BioLegend	103032
**CD127**	SB/199	PE	BioLegend	121111
**CD69**	H1.2F3	PE-Cy7	BioLegend	104512
**MHC-I multimer**		APC		
**CD4**	GK1.5	APC-Cy7	BioLegend	100414

Supplementary Table 4. List of antibodies used for myeloid cells staining analyzed on BD FACS Canto II instrument

Antigen	Clone	Fluorochrome	Company	Catalog#
CD45	30-F11	BV421	Biolegend	103134
Ly6G	1A8	APC/Cy7	Biolegend	127651
CD11c	N418	PE	Biolegend	117307
CD8a	53-6.7	BV570	Biolegend	100740
CD19	6D5	BV570	Biolegend	115535
Ly6C	HK1.4	PE/Cy7	Biolegend	128018
CD11b	M1/70	FITC	Biolegend	101205
IAIE	M5/114.15.2	Percp/Cy5.5	Biolegend	107626
F4/80	BM8	APC	Biolegend	123116

Supplementary Table 5. List of antibodies used for tumor cells phenotyping on BD Fortessa instrument

Antigen	Clone	Fluorochrome	Company	Catalog#
**CD103**	M290	BUV496	BD OptiBuild™	741083
**CD11b**	M1/70	BUV395	BD Horizon™	563553
**CD45**	30-F11	BV605	BD Horizon™	563053
**Live/Dead**	N/a	Live-Dead Yellow	Life Technologies	L34959
**PD-L1**	10F.9G2	BV421	BioLegend	124315
**Fas**	SA367H8	PerCP-Cy5.5	BioLegend	152610
**H-2**	M1/42	FITC	BioLegend	125508
**Rae1-γ**	CX1	PE	BioLegend	130107
**H-2D^b**	KH95	APC-Fire 750	BioLegend	111519
**CRT**	1G6A7	APC	NOVUS	NBP1-47518APC

Supplementary Table 6. List of antibodies used for intracellular cytokine staining analyzed on BD FACS Canto II instrument

Antigen	Clone	Fluorochrome	Company	Catalog#
**CD45**	30-F11	BV421	Biolegend	103134
**CD4**	RM4-5	APC/Cy7	Biolegend	100526
**CD8**	53-6.7	PE	Biolegend	100707
**Live/Dead**	N/a	Live-Dead Yellow	Life Technologies	L34959
**CD44**	IM7	PE/Cy7	Biolegend	103030
**IFN-γ**	XMG1.2	FITC	Biolegend	505806
**IL-2**	JES6-1A12	Percp/Cy5.5	Biolegend	503821
**TNF-α**	MP6-XT22	APC	Biolegend	506307
